# Associations between serum biomarkers of fruit and vegetable intake and all-cause, cancer and CVD mortality among US adults

**DOI:** 10.1017/S000711452510531X

**Published:** 2025-11-28

**Authors:** Kijoon Kim, Seoeun Ahn, Matthew P. Madore, Ock K. Chun

**Affiliations:** 1 Department of Nutritional Sciences, University of Connecticut, Storrs, CT 06269, USA; 2 Kim Kijoon BOM Clinic, Seoul 05554, South Korea

**Keywords:** Fruit and vegetable, Serum biomarker, Vitamin C, Potassium, Carotenoids, Mortality, NHANES

## Abstract

Using serum biomarkers that reflect fruit and vegetable (FV) intake offers a significant advantage over traditional dietary assessments by providing a more objective, accurate measure, meaningfully minimising recall bias and misreporting common in self-reported dietary data. This study investigated the relationship between these serum biomarkers and mortality risk using data from 19 168 adults aged 30 years and older who participated in the National Health and Nutrition Examination Survey from 1988 to 2006. Mortality follow-up was determined by linkage to the National Death Index through 31 December 2019 and diet by 24-h recalls. Cox proportional hazards models were employed to calculate hazard ratios (HR) and 95 % CI for mortality outcomes by tertiles of serum biomarkers of FV intake. Higher serum concentrations of total carotenoids were associated with a reduced risk of all-cause (tertile 3 *v*. tertile 1 HR = 0·69, 95 % CI = 0·61, 0·78) and cancer mortality (HR = 0·53, 95 % CI = 0·39, 0·71). Greater serum concentrations of individual carotenoids, such as *α*-carotene, *β*-carotene, *β*-cryptoxanthin, lycopene, lutein and zeaxanthin, were also linked to lower risks of all-cause and cancer mortality. Higher serum potassium concentrations showed a trend towards an association with a greater risk of all-cause mortality. No significant associations were found between serum vitamin C concentrations and mortality outcomes in the overall analysis; however, in sex-stratified analyses, higher vitamin C concentrations were associated with reduced risk of all-cause and cancer mortality in women. These findings suggest that specific serum biomarkers of FV intake, particularly carotenoids and vitamin C, may serve as indicators of reduced mortality risk.

Numerous epidemiological studies have established that greater consumption of fruits and vegetables (FV) is associated with a reduced risk of chronic diseases, including CVD, heart disease and cancer^(1–3)^. FV are rich in a variety of phytochemicals, including carotenoids and flavonoids, which are believed to contribute to their health benefits^(4)^. However, much of the existing evidence is based on self-reported dietary data, which is often prone to inaccuracies and subject to recall and social desirability biases. These limitations underscore the need for objective measures of dietary intake to establish more reliable associations with health outcomes.

Serum biomarkers, such as vitamin C, potassium and carotenoids, provide an objective and quantifiable measure of FV intake^(5,6)^. Among these, carotenoids are naturally occurring pigments found in a wide range of FV. However, since humans cannot synthesis carotenoids, blood carotenoid levels are considered one of the best biomarkers of FV intake^(7)^. Additionally, carotenoids function as antioxidants and some are vitamin A precursors, underscoring their significant role in promoting human health. Epidemiological studies have reported that higher intake of carotenoids is associated with improved immune function^(8)^, anti-inflammatory effects^(9)^ and enhanced cognitive function^(10)^, as well as a reduced risk of chronic diseases such as CVD, cancer and age-related eye diseases^(11–13)^. Vitamin C, an antioxidant found abundantly in FV, plays a crucial role in immune function, collagen synthesis and protection against oxidative stress^(14)^. Since vitamin C is rapidly absorbed and is largely excreted when consumed in excess, it is expected to reflect short-term FV intake^(15)^. Potassium, another nutrient abundant in FV, is essential for maintaining normal blood pressure, muscle and renal function and cardiovascular health^(16,17)^. Although potassium is rich in FV, its levels are constantly regulated under normal health conditions^(18)^, making it challenging to capture longer-term FV intake.

CVD and cancers are the leading causes of death worldwide, highlighting the importance of identifying dietary factors that could mitigate these risks. Despite the well-recognised benefits of FV consumption, few studies have utilised serum biomarkers to explore their relationship with cause-specific mortality in a large, nationally representative dataset. Our analytic dataset combines publicly available National Health and Nutrition Examination Survey (NHANES) data with National Death Index-linked mortality files. This study aims to elucidate the relationships between serum carotenoids, vitamin C and potassium and all-cause, cancer and CVD mortality among US adults aged 30 years and older using data from the NHANES spanning 1988–2006 with mortality follow-up through 2019.

## Materials and methods

### Study population

**Figure 1.**
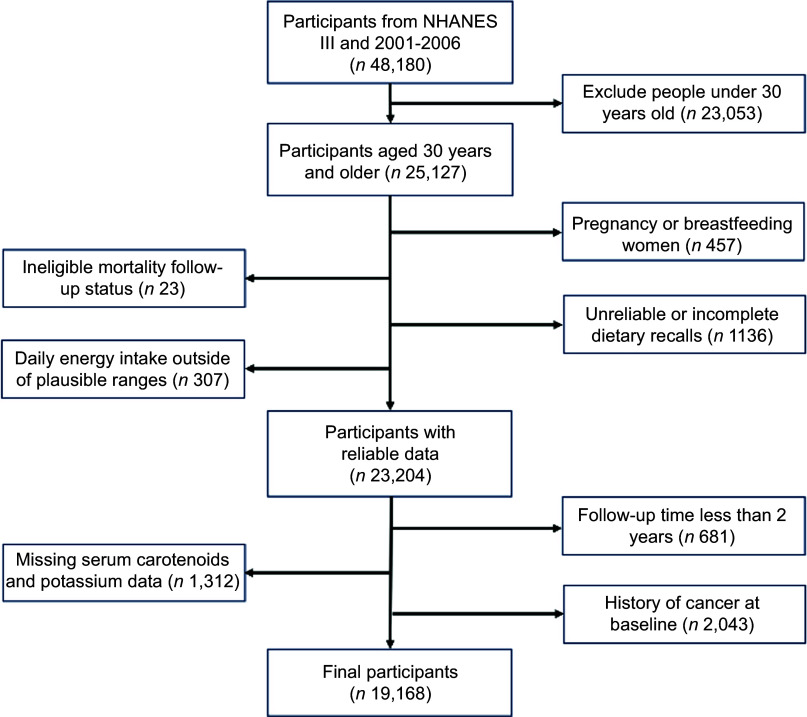
Flow chart of the study population (NHANES III and 2001–2006).

This study utilised data from the NHANES for US adults aged 30 years and older, spanning the periods 1988–1994 (*n* 13 065)^(19)^ and 2001–2006 (*n* 12 062)^(20–22)^. The combined dataset included a total of 25 127 participants. We focused on participants aged 30 years and older to avoid bias, as mortality due to chronic diseases is more prevalent in this age group, and deaths under the age of 30 are often due to external factors^(23)^. Participants were excluded if they met any of the following criteria: pregnancy or breast-feeding women (*n* 457), ineligible mortality follow-up status (*n* 23), unreliable or incomplete dietary recalls (*n* 1136) and daily energy intake outside of plausible ranges (< 500 or > 8000 kcal/d for men and < 500 or > 5000 kcal/d for women) (*n* 307). Additional exclusions were applied to individuals with less than 2 years of follow-up time (*n* 681), missing data on serum carotenoids and potassium levels (*n* 1312) or with a history of cancer at baseline (*n* 2043). As a result, we utilised data from 19 168 participants for the analysis (Fig. 1). NHANES study protocols were approved by the National Center for Health Statistics research ethics review board (Institutional Review Board approval and documented consent from participants for NHANES 1988–1994, Protocol #98-12 for NHANES 2001–2004 and Protocol #2005-06 for NHANES 2005–2006). This secondary analysis used publicly available, de-identified NHANES and National Death Index public-use linked mortality files and, per our institutional policies, did not require additional IRB review.

### Estimation of dietary intake of carotenoids, vitamin C and potassium

Dietary intake of carotenoids was estimated using one-day 24-h dietary recalls collected from NHANES 1988–1994 to 2001–2006. For vitamin C and potassium, we used intake data as calculated and provided by NHANES. The estimation of carotenoid intake was performed using NHANES dietary data in conjunction with the USDA’s Food and Nutrient Database for Dietary Studies (FNDDS) versions 1.0^(24)^, 2.0^(25)^ and 3.0^(26)^. The FNDDS serves as a resource for determining nutrient values in NHANES, drawing on data from the USDA National Nutrient Database for Standard Reference. The FNDDS provides details on the linkage between NHANES food items and standard reference entries, allowing for the estimation of carotenoid intake by summing the carotenoid content of each food item reported in the dietary recall. By using the FNDDS database, which includes detailed recipes for various foods, we were able to identify the carotenoid content of individual components in mixed dishes and aggregate these values to estimate the total and individual carotenoid intake from each food source. This approach allows for a comprehensive assessment of dietary intake by accurately capturing the carotenoid content in both whole foods and complex dishes. This calculation encompassed specific carotenoids, including *α*-carotene, *β*-carotene, *β*-cryptoxanthin, lycopene, lutein and zeaxanthin and total carotenoid consumption. Additionally, the dietary intake estimates for vitamin C and potassium were similarly derived, using FNDDS nutrient profiles to ensure accurate and reliable calculations for these essential nutrients.

### Analysis of serum carotenoids, vitamin C and potassium

Serum concentrations of *α*-carotene, *β*-carotene, *β*-cryptoxanthin, lycopene and lutein and zeaxanthin were analysed using HPLC^(27,28)^. Serum vitamin C was quantified using isocratic HPLC with electrochemical detection at 650 mV. Serum potassium levels were determined using an ion-selective electrode method. The ion-selective electrode method utilises a selective membrane that measures potassium ion concentration by detecting the potential difference across the membrane, ensuring precise and specific detection of serum potassium levels. Serum carotenoids were assayed by HPLC in both NHANES III (1988–1994) and NHANES 2001–2006; for 2003–2004, a comparable HPLC method at Craft Technologies was calibrated to the CDC method using CDC-published Deming regression equations to ensure cross-cycle comparability.

### National Health and Nutrition Examination Survey linked mortality data

For mortality studies, longer-term datasets and larger sample sizes are essential. Therefore, we combined two public-use NHANES-linked mortality datasets from 1988–1994 to 2001–2006^(29)^. The National Center for Health Statistics has linked publicly available data collected from several National Center for Health Statistics population surveys with death certificate records from the National Death Index. Follow-up time for each participant was calculated from the date of the interview until the date of death or censoring. NHANES-linked mortality data provide follow-up information from the date of survey participation through 31 December 2019. The causes of death for deaths occurring prior to 1999 were determined using the 9th revision of the International Statistical Classification of Diseases, Injuries, and Causes of Death (ICD-9) guidelines^(30)^. For deaths occurring after 1998, the 10th revision (ICD-10) guidelines^(31)^ were used.

### Covariates

Covariates were selected based on previous literature, prioritising variables commonly used in similar studies. Race/ethnicity was coded as non-Hispanic White, non-Hispanic Black and Mexican American (NHANES public-use categories). Total energy intake and saturated fatty acid intake were derived from the 24-h dietary recall. Diabetes and hypertension were defined by self-reported physician diagnosis and/or medication use; hypertension was also identified when measured systolic BP ≥ 140 mmHg or diastolic BP ≥ 90 mmHg. History of CVD (coronary heart disease, myocardial infarction or stroke) was based on self-report. Aspirin use and dietary supplement use were self-reported current use at baseline. ‘Examination season’ (warm (May–October) *v*. cool (November–April)) was used only in sensitivity analyses. Smokers were classified as current smokers if they had smoked at least 100 cigarettes in their lifetime and currently smoked on some days or every day. Non-smokers were defined as those who had smoked fewer than 100 cigarettes in their lifetime, and former smokers were those who had smoked at least 100 cigarettes in their lifetime but had quit by the time of the interview. Alcohol consumption was categorised as no consumption (0 drinks), moderate consumption (up to two drinks per day for men and up to one drink per day for women) or heavy consumption (more than two drinks per day for men and more than one drink per day for women), based on participants’ self-reported daily intake of any alcoholic beverages^(32)^. Physical activity was measured as the metabolic equivalent of tasks, calculated from weekly minutes of walking/bicycling and moderate/vigorous recreational activities. Metabolic equivalent of tasks-minutes per week were calculated by multiplying weekly minutes of activity by the assigned metabolic equivalent of task values. Participants who reported no walking/bicycling or moderate/vigorous recreational activities were classified as inactive. Poverty income ratio (PIR) was calculated as the ratio of family income to the poverty threshold. Participants were also categorised based on their PIR into three groups: PIR < 1·3, 1·3 ≤ PIR < 1·85, and PIR ≥ 1·85. Chronic kidney disease (CKD) was included as a covariate, categorised as ‘Yes’ or ‘No,’ based on the estimated glomerular filtration rate. We calculated estimated glomerular filtration rate using the 2021 CKD-EPI creatinine equation without race adjustment^(33)^. Individuals with estimated glomerular filtration rate less than 60 ml/min/1·73 m^2^ were classified as CKD ‘Yes’, and those with estimated glomerular filtration rate equal to or greater than 60 ml/min/1·73 m² were classified as CKD ‘No’. This categorisation was used to account for the role of kidney function in regulating potassium levels and its potential impact on mortality risk, acknowledging that CKD may influence these associations. Covariates for cause-specific mortality were selected based on previous studies^(34–36)^. Participants with missing covariate data were automatically excluded from analyses when relevant data were unavailable.

### Statistical analysis

We compared serum concentrations of vitamin C, potassium and total and individual carotenoids according to participants’ baseline socio-demographic and health-related characteristics. Additionally, we compared dietary intakes of fruits, vegetables, vitamin C, potassium and carotenoids by categorising participants into tertiles based on their serum concentrations of vitamin C, potassium and total carotenoids. Given that NHANES collects data cross-sectionally without repeated measures, and that single biomarker measurements may lead to regression dilution over time, we evaluated the potential impact of this dilution by calculating HR and 95 % CI for all-cause mortality across different follow-up periods: less than 5 years, 5 to less than 10 years, 10 to less than 15 years and 15 years or more. These categories correspond to the quartiles of the overall person-time distribution. In this analysis, participants with follow-up periods of less than 2 years were excluded. We also performed stratified analyses according to sex, baseline BMI and smoking status.

All statistical analyses were conducted using SAS software, version 9.4 (SAS Institute Inc.), employing SAS PROC SURVEY procedures and incorporating the appropriate weights, strata, domain and cluster variables to account for the complex, multistage probability sampling design. Cox proportional hazards models were employed to calculate multivariable-adjusted hazard ratios (HR) and 95 % CI for all-cause, cancer and CVD mortality according to tertiles of serum carotenoids, vitamin C and potassium. Two models were fitted: model 1 adjusted for age, sex and race/ethnicity and model 2 additionally adjusted for energy intake, BMI, PIR, diabetes, hypertension, alcohol consumption, saturated fatty acid intake, aspirin use, supplement use, CKD and history of CVD (excluded in CVD-mortality models). Total energy intake was included to obtain an isocaloric interpretation and to reduce confounding by overall diet quantity and BMI to account for adiposity-related confounding. In additional analyses, we assessed temporal heterogeneity by testing biomarker × survey–period interactions (NHANES III 1988–1994 *v*. 2001–2006) in fully adjusted models. We also conducted a sensitivity analysis by adding 24-h dietary intakes of fruits, vegetables, vitamin C, potassium, total carotenoids and examination season to the fully adjusted model and assessed multicollinearity, to evaluate whether biomarker–mortality associations were independent of concurrent reported diet and seasonality. All reported *P* values are two sided with a significance level of *α* = 0·05.

## Results

Table 1 presents the serum concentrations of vitamin C, potassium and carotenoids across various socio-demographic and health-related characteristics. Generally, higher serum concentrations of vitamin C and carotenoids were observed in older participants, females, those with normal weight, never smokers, moderate drinkers, those engaging in vigorous physical activity, individuals with a higher PIR, those without a history of diabetes mellitus, CHD or stroke and supplement users.

**Table 1.** Mean serum concentrations of vitamin C, potassium and carotenoids by sociodemographic and health-related characteristics in USA. Adults aged ≥ 30 years participating in NHANES 1988–1994 and 2001–2006*

	*n*	%	Vitamin C^†^		Potassium		Total carotenoid		*α*-carotene		*β*-carotene		*β*-cryptoxanthin		Lycopene		Lutein and zeaxanthin	
			(µmol/l)		(mmol/l)		(µmol/l)		(µmol/l)		(µmol/l)		(µmol/l)		(µmol/l)		(µmol/l)	
			Mean	se	Mean	se	Mean	se	Mean	se	Mean	se	Mean	se	Mean	se	Mean	se
All	19 168		48·0	0·5	4·04	0·01	1·55	0·01	0·09	0·00	0·39	0·01	0·17	0·00	0·57	0·01	0·34	0·00
Sex																		
Male	9360	48·8	43·5	0·6	4·09	0·01	1·50	0·01	0·08	0·00	0·32	0·01	0·16	0·00	0·60	0·01	0·33	0·00
Female	9808	51·2	52·4	0·7	4.00	0·01	1·61	0·02	0·10	0·00	0·45	0·01	0·18	0·00	0·54	0·01	0·34	0·00
* P* value			<0·0001		<0·0001		<0·0001		<0·0001		<0·0001		<0·0001		<0·0001		0·14	
Age (years)																		
<50	9010	56·5	44·2	0·7	4·02	0·01	1·51	0·01	0·09	0·00	0·34	0·01	0·16	0·00	0·60	0·01	0·32	0·00
50 to < 70	6541	32·3	51·1	0·7	4·06	0·01	1·61	0·02	0·10	0·00	0·42	0·01	0·17	0·00	0·57	0·01	0·35	0·00
≥ 70	3617	11·2	58·5	0·8	4·15	0·01	1·61	0·02	0·10	0·00	0·53	0·01	0·18	0·00	0·43	0·01	0·37	0·01
* P* value			<0·0001		<0·0001		<0·0001		<0·0001		<0·0001		<0·0001		<0·0001		<0·0001	
Ethnicity																		
Non-Hispanic White	9029	78·4	49·3	0·6	4·07	0·01	1·54	0·02	0·09	0·00	0·39	0·01	0·16	0·00	0·58	0·01	0·32	0·00
Non-Hispanic Black	4583	10·6	41.0	0·6	3·97	0·01	1·54	0·02	0·08	0·00	0·37	0·01	0·17	0·00	0·56	0·01	0·37	0·01
Mexican-American	4487	5·8	45·6	0·8	3·98	0·01	1·62	0·02	0·09	0·00	0·35	0·01	0·28	0·01	0·55	0·63	0·35	0·01
Others	644	5·2	52·9	1·8	3·97	0·02	1·66	0·05	0·10	0·01	0·39	0·02	0·22	0·01	0·63	0·03	0·33	0·01
* P* value			<0·0001		<0·0001		<0·01		<0·01		<0·01		<0·0001		<0·05		<0·0001	
BMI (kg/m^2^)																		
<25	5888	34·3	51.0	0·7	4·05	0·01	1·71	0·02	0·11	0·00	0·49	0·01	0·19	0·00	0·55	0·01	0·37	0·01
25 to < 30	7074	35·6	49·1	0·8	4·05	0·01	1·58	0·01	0·09	0·00	0·38	0·01	0·17	0·00	0·58	0·01	0·35	0·00
≥ 30	5981	30·1	43·4	0·7	4·03	0·01	1·36	0·02	0·07	0·00	0·28	0·01	0·14	0·00	0·58	0·01	0·29	0·00
* P* value			<0·0001		<0·05		<0·0001		<0·0001		<0·0001		<0·0001		<0·0001		<0·0001	
Smoking^‡^																		
Never	9193	47·4	53·1	0·6	4·01	0·01	1·69	0·02	0·11	0·00	0·45	0·01	0·19	0·00	0·59	0·01	0·35	0·00
Former	5382	27·6	51·2	0·7	4·08	0·01	1·60	0·02	0·09	0·00	0·42	0·01	0·17	0·00	0·56	0·01	0·36	0·01
Current	4583	25·0	35·3	1·0	4·08	0·01	1·26	0·01	0·05	0·00	0·25	0·01	0·12	0·00	0·55	0·01	0·29	0·00
* P* value			<0·0001		<0·0001		<0·0001		<0·0001		<0·0001		<0·0001		<0·0001		<0·0001	
Alcohol consumption^§^																		
None	11 000	51·5	48·4	0·9	4·05	0·01	1·49	0·02	0·09	0·00	0·40	0·01	0·17	0·00	0·49	0·01	0·33	0·00
Moderate	3606	23·8	51·5	0·7	4·05	0·01	1·74	0·02	0·10	0·00	0·43	0·01	0·18	0·00	0·68	0·01	0·36	0·01
Heavy	4277	24·7	44·4	0·9	4·03	0·01	1·50	0·02	0·08	0·00	0·31	0·01	0·15	0·00	0·64	0·01	0·33	0·01
* P* value			<0·0001		<0·05		<0·0001		<0·0001		<0·0001		<0·0001		<0·0001		<0·0001	
Physical activity^||^																		
Sedentary	1269	8·6	41.0	1·5	4·01	0·01	1·41	0·03	0·08	0·00	0·32	0·01	0·15	0·00	0·53	0·01	0·33	0·01
Moderate	1641	10·9	43·5	1·2	4·05	0·01	1·46	0·03	0·08	0·00	0·34	0·01	0·15	0·00	0·56	0·01	0·32	0·01
Vigorous	10 152	80·4	50·6	0·7	4·05	0·01	1·65	0·02	0·10	0·00	0·43	0·01	0·18	0·00	0·58	0·01	0·36	0·00
* P* value			<0·0001		0·056		<0·0001		<0·0001		<0·0001		<0·0001		<0·001		<0·0001	
PIR^¶^																		
<1·3	4970	16·3	40·3	0·8	4·04	0·01	1·38	0·02	0·07	0·00	0·33	0·01	0·16	0·00	0·5	0·01	0·32	0·01
1·3 to < 1·85	2325	9·6	44·4	0·9	4·05	0·01	1·45	0·03	0·08	0·00	0·36	0·01	0·16	0·00	0·52	0·01	0·33	0·01
≥ 1·85	10 508	74·1	50·3	0·6	4·04	0·01	1·61	0·01	0·09	0·00	0·40	0·01	0·17	0·00	0·60	0·01	0·34	0·00
* P* value			<0·0001		0·43		<0·0001		<0·0001		<0·0001		<0·01		<0·0001		<0·01	
Diabetes mellitus**																		
Yes	2132	10·4	42·6	1·0	4·09	0·01	1·36	0·02	0·07	0·00	0·31	0·01	0·15	0·00	0·49	0·01	0·34	0·01
No	12 803	89·6	45·8	0·7	4·07	0·01	1·54	0·01	0·09	0·00	0·40	0·01	0·17	0·00	0·53	0·01	0·35	0·00
* P* value			<0·001		0·15		<0·0001		<0·0001		<0·0001		<0·0001		<0·01		0·076	
Hypertension^††^																		
Yes	7706	33·9	49·3	0·8	4·03	0·01	1·49	0·02	0·08	0·00	0·37	0·01	0·16	0·00	0·54	0·01	0·34	0·01
No	10 979	66·1	47·5	0·6	4·05	0·01	1·59	0·01	0·10	0·00	0·40	0·01	0·17	0·00	0·59	0·01	0·34	0·00
* P* value			<0·05		<0·05		<0·0001		<0·0001		<0·01		<0·0001		<0·0001		0·14	
Aspirin use^‡‡^																		
Yes	2238	11	50·5	1·2	4·12	0·01	1·49	0·02	0·09	0·00	0·40	0·01	0·16	0·00	0·50	0·01	0·35	0·01
No	16 930	89	47·8	0·5	4·04	0·01	1·56	0·01	0·09	0·00	0·39	0·01	0·17	0·00	0·58	0·01	0·34	0·00
* P* value			<0·05		<0·0001		<0·01		<0·05		0·425		<0·05		<0·0001		0·21	
Supplement use^§§^																		
Yes	8633	49·9	58·8	0·6	4·04	0·01	1·69	0·02	0·10	0·00	0·48	0·01	0·18	0·00	0·59	0·01	0·34	0·00
No	10 535	50·1	37·7	0·6	4·05	0·01	1·42	0·01	0·08	0·00	0·30	0·01	0·16	0·00	0·55	0·01	0·33	0·00
* P*-value			<0·0001		0·15		<0·0001		<0·0001		<0·0001		<0·0001		<0·0001		0·18	
History of CHD or stroke																		
Yes	1192	4·2	47·3	1·5	4·12	0·02	1·38	0·03	0·08	0·00	0·36	0·01	0·15	0·01	0·47	0·02	0·32	0·01
No	17 976	95·8	48·1	0·6	4·04	0·01	1·56	0·01	0·09	0·00	0·39	0·01	0·17	0·00	0·57	0·01	0·34	0·00
* P* value			0·60		<0·0001		<0·0001		<0·001		<0·05		<0·01		<0·0001		0·064	
CKD^||||^																		
Yes	3081	10·3	50·2	0·77	4·16	0·01	1·58	0·02	0·09	0·00	0·49	0·01	0·18	0·00	0·43	0·01	0·40	0·01
No	16 520	89·7	47·7	0·55	4·03	0·01	1·55	0·01	0·09	0·00	0·38	0·01	0·17	0·00	0·58	0·01	0·33	0·00
* P* value			<0·001		<0·0001		0·12		<0·05		<0·0001		<0·001		<0·0001		<0·0001	

*Values are means (se). ^†^For serum vitamin C, data from 15 716 participants in the 2001 to 2004 NHANES were used. ^‡^Smokers were defined as current smokers if they had smoked at least 100 cigarettes in their lifetime and smoked some days or every day, non-smokers if they had smoked fewer than 100 cigarettes in their lifetime and former smokers if they had smoked at least 100 cigarettes in their lifetime but had reported quitting by the time of the interview. ^§^Alcohol consumption was defined as either no consumption (0 drinks), moderate consumption (no more than two drinks/day for men and no more than one drink/day for women) or heavy consumption (more than two drinks/day for men and more than one drink/day for women), based on the number of drinks of any type of alcoholic beverage participants reported drinking per day. ^||^Physical activity level, expressed using the metabolic equivalent of task score, was calculated by combining the intensity level of leisure time activities reported mean duration, and frequency. ^¶^Poverty income ratio. **Diabetes medication included insulin and/or medication to lower blood sugar. ^††^Hypertension was defined as systolic blood pressure exceeding 140 mmHg or diastolic pressure over 90 mmHg or taking prescribed medicine for high blood pressure. ^‡‡^Aspirin users defined as those using aspirin regularly during the past month. ^§§^Supplement use defined as those using nutritional supplements during the past month. ^||||^CKD classified as ‘Yes’ for eGFR < 60 ml/min/1·73 m² ‘No’ for eGFR ≥ 60 ml/min/1·73 m².

In contrast, serum potassium concentrations exhibited a somewhat different pattern. Serum potassium concentrations were higher in males, former or current smokers, supplement non-users, those with a history of CHD or stroke and CKD compared with their counterparts.

Averages of dietary intakes of fruits, vegetables, vitamin C, potassium and carotenoids across tertiles of serum concentrations of vitamin C, potassium and carotenoids are presented in Table 2. As serum concentrations of vitamin C and total carotenoids increased, dietary intakes of fruits, vegetables, vitamin C, potassium and total and individual carotenoids significantly increased (*P*_for trend_ < 0·01 for all). However, as serum potassium concentrations increased, dietary intakes of vegetables, vitamin C, potassium and lycopene increased, but not fruits or other carotenoids.

**Table 2.** Average intake of fruit, vegetable, vitamin C, potassium and carotenoids by tertiles of serum concentrations of vitamin C, potassium and total carotenoids among US adults aged ≥ 30 years participating in NHANES 1988–1994 and 2001–2006*

	Serum vitamin C^†^							Serum potassium^‡^							Total serum carotenoids^§^						
	T1(*n* 5271)		T2(*n* 5174)		T3(*n* 5271)		*P*_for trend_^||^	T1(*n* 5857)		T2(*n* 6953)		T3(*n* 6358)		*P*_for trend_^||^	T1 (*n* 6389)		T2(*n* 6390)		T3(*n* 6389)		*P*_for trend_^||^
	Mean	se	Mean	se	Mean	se		Mean	se	Mean	se	Mean	se		Mean	se	Mean	se	Mean	se	
Fruits (g/d)	66·0	2·9	160	5	228	7	<0·0001	153	5	157	4	170	5	0·054	105	4	156	4	216	6	<0·0001
Vegetables (g/d)	180	4	214	5	221	4	<0·0001	191	5	204	4	215	4	<0·05	170	3	203	4	237	4	<0·0001
Fruits and vegetables (g/d)	246	5	373	6	448	7	<0·0001	344	7	361	6	386	6	<0·01	275	5	358	5	453	7	<0·0001
Vitamin C (mg/d)	62·9	1·8	98·2	2·5	121	3	<0·0001	91·2	1·8	95·5	2·0	99·3	2·3	<0·01	73·6	1·6	95·5	2·2	116	2	<0·0001
Potassium (mg/d)	2694	35	2912	26	2939	24	<0·0001	2660	27	2848	23	3001	27	<0·0001	2609	22	2871	27	3037	23	<0·0001
Total carotenoids (mcg/d)	8028	265	10 050	285	10 244	237	<0·0001	8817	262	9959	232	9622	242	0·2	6855	162	9302	247	12 177	237	<0·0001
*α*-carotene (mcg/d)	285	16	438	26	511	24	<0·0001	405	20	433	19	431	20	0·57	305	16	387	20	572	23	<0·0001
*β*-carotene (mcg/d)	1563	54	2263	91	2620	72	<0·0001	2188	75	2197	62	2209	69	0·85	1560	49	1999	60	2987	73	<0·0001
*β*-cryptoxanthin (mcg/d)	58·3	2·4	112	4	158	6	<0·0001	115	4	113	3	124	4	0·33	78·4	2·5	116	4	155	5	<0·0001
Lycopene (mcg/d)	4942	238	5555	182	5188	204	<0·01	4491	167	5695	196	5314	187	<0·05	3724	155	5326	199	6485	197	<0·0001
Lutein and zeaxanthin (mcg/d)	1180	48	1682	96	1768	50	<0·0001	1618	80	1522	48	1544	60	0·33	1188	55	1476	49	1978	60	<0·0001

*Values are mean (se). Serum concentration ranges for each nutrient: ^†^(0–268·0 µmol/l). ^‡^(2·5–6·2 mmol/l). ^§^(0–15·0 µmol/l). ^||^Adjusted for age, gender and ethnicity.

Hazard ratios and 95 % CI for all-cause, cancer and CVD mortality across tertiles of serum concentrations of vitamin C, potassium and total and individual carotenoids are presented in Table 3. All biomarker × period interaction tests were non-significant after adjustment for multiple comparisons; thus, pooled hazard ratios are presented as primary results. During a mean follow-up of 18·8 years, 7186 all-cause deaths, 1509 cancer deaths and 2537 CVD deaths were identified. In age, sex and ethnicity adjusted model (model 1), higher serum concentrations of vitamin C, total carotenoids, *α*-carotene, *β*-carotene, *β*-cryptoxanthin, lycopene and lutein and zeaxanthin were associated with all-cause, cancer and CVD mortality. After further adjustment for potential confounders in model 2, the inverse associations between serum concentrations of total carotenoids, *α*-carotene, *β*-carotene, *β*-cryptoxanthin, lycopene and lutein and zeaxanthin and all-cause and cancer mortality remained significant. The associations between serum concentrations of *β*-carotene and *β*-cryptoxanthin and CVD mortality also remained marginally significant. However, while the association between serum vitamin C and cancer mortality remained significant in model 2, the association between serum vitamin C and all-cause and CVD mortality was attenuated and no longer statistically significant. Conversely, while the association was no longer statistically significant in model 2, a higher serum potassium concentration showed an increasing trend with all-cause mortality.

**Table 3.** Multivariable HRs and 95 % CI for all-cause, cancer and CVD mortality by tertiles of serum concentrations of vitamin C, potassium and total and individual carotenoids among US adults aged ≥ 30 years participating in NHANES 1988–2006

		All-cause mortality					Cancer mortality					CVD mortality				
	Person-years	*n* of death/n	Model 1*		Model 2^†^		*n* of death/n	Model 1*		Model 2^†^		*n* of death/n	Model 1*		Model 2^†^	
			HR	95 % CI	HR	95 % CI		HR	95 % CI	HR	95 % CI		HR	95 % CI	HR	95 % CI
Vitamin C^‡^																
T1	102 312	2350/5271	1·00			1·00	567/5271	1·00			1·00	795/5271	1·00		1·00	
T2	101 678	1829/5174	0·69	0·62, 0·77	0·89	0·78, 1·02	370/5174	0·53	0·43, 0·66	0·71	0·55, 0·93	655/5174	0·76	0·64, 0·89	0·92	0·70, 1·20
T3	91 113	1962/5271	0·60	0·54, 0·66	0·91	0·80, 1·04	370/5271	0·56	0·46, 0·68	0·74	0·56, 0·97	747/5271	0·61	0·52, 0·71	0·91	0·70, 1·17
* P*_for trend_			<0·0001			0·19		<0·0001			<0·05		<0·0001		0·45	
Potassium^§^																
T1	110 455	1964/5857	1·00			1·00	422/5857	1·00			1·00	703/5857	1·00		1·00	
T2	131 222	2307/6953	0·95	0·86, 1·04	0·97	0·85, 1·10	490/6953	0·91	0·75, 1·11	0·89	0·68, 1·18	801/6953	0·94	0·81, 1·08	1·07	0·86, 1·32
T3	109 040	2915/6358	1·14	1·06, 1·24	1·10	0·99, 1·23	597/6358	1·16	0·97, 1·37	1·01	0·76, 1·36	1033/6358	1·10	0·96, 1·25	1·18	0·97, 1·43
* P*_for trend_			<0·001			<0·05		0·06			0·82		0·12		0·07	
Total carotenoids^||^																
T1	114 383	2868/6389	1·00			1·00	650/6389	1·00			1·00	934/6389	1·00		1·00	
T2	120 821	2202/6390	0·65	0·61, 0·70	0·74	0·66, 0·83	456/6390	0·62	0·52, 0·72	0·72	0·58, 0·89	804/6390	0·74	0·64, 0·85	0·85	0·68, 1·06
T3	115 514	2116/6389	0·51	0·47, 0·55	0·69	0·61, 0·78	403/6389	0·46	0·38, 0·54	0·53	0·39, 0·71	799/6389	0·59	0·52, 0·68	0·89	0·72, 1·10
* P*_for trend_			<0·0001			<0·0001		<0·0001			<0·0001		<0·0001		0·27	
*α*-carotene^¶^																
T1	115 959	2594/6576	1·00			1·00	588/6576	1·00			1·00	863/6576	1·00		1·00	
T2	109 498	2206/6037	0·67	0·62, 0·73	0·86	0·77, 0·96	455/6037	0·75	0·64, 0·87	0·97	0·77, 1·22	791/6037	0·69	0·60, 0·80	0·82	0·66, 1·02
T3	125 261	2386/6555	0·51	0·47, 0·56	0·75	0·66, 0·86	466/6555	0·51	0·44, 0·61	0·74	0·57, 0·96	883/6555	0·55	0·48, 0·64	0·85	0·66, 1·08
* P*_for trend_			<0·0001			<0·0001		<0·0001			<0·05		<0·0001		0·22	
*β*-carotene**																
T1	118 167	2295/6434	1·00			1·00	554/6434	1·00			1·00	744/6434	1·00		1·00	
T2	117 118	2264/6311	0·66	0·60, 0·72	0·77	0·67, 0·88	461/6311	0·65	0·54, 0·77	0·86	0·66, 1·13	816/6311	0·71	0·59, 0·85	0·79	0·60, 1·05
T3	115 432	2627/6423	0·54	0·49, 0·59	0·71	0·64, 0·80	494/6423	0·53	0·44, 0·63	0·66	0·51, 0·87	977/6423	0·56	0·48, 0·66	0·81	0·66, 1·01
* P*_for trend_			<0·0001			<0·0001		<0·0001			<0·01		<0·0001		0·07	
*β*-cryptoxanthin^††^																
T1	99 434	2502/5886	1·00			1·00	575/5886	1·00			1·00	846/5886	1·00		1·00	
T2	126 051	2490/6692	0·72	0·66, 0·79	0·84	0·75, 0·94	538/6692	0·62	0·52, 0·74	0·81	0·64, 1·01	863/6692	0·77	0·67, 0·89	0·86	0·72, 1·03
T3	125 232	2194/6590	0·53	0·48, 0·58	0·72	0·63, 0·83	396/6590	0·42	0·34, 0·52	0·60	0·44, 0·82	828/6590	0·62	0·54, 0·71	0·84	0·69, 1·03
* P*_for trend_			<0·0001			<0·0001		<0·0001			<0·001		<0·0001		0·083	
Lycopene^‡‡^																
T1	113 765	3491/6384	1·00			1·00	690/6384	1·00			1·00	1258/6384	1·00		1·00	
T2	126 686	2240/6401	0·75	0·70, 0·81	0·83	0·75, 0·92	484/6401	0·72	0·61, 0·84	0·70	0·55, 0·88	766/6401	0·78	0·69, 0·88	0·88	0·71, 1·10
T3	110 266	1455/6383	0·64	0·58, 0·70	0·77	0·68, 0·88	335/6383	0·64	0·54, 0·75	0·73	0·57, 0·93	513/6383	0·67	0·58, 0·77	0·85	0·69, 1·06
* P*_for trend_			<0·0001			<0·0001		<0·0001			<0·01		<0·0001		0·12	
Lutein and zeaxanthin^§§^																
T1	106 774	2280/6397	1·00			1·00	488/6397	1·00			1·00	747/6397	1·00		1·00	
T2	118 186	2259/6381	0·73	0·68, 0·79	0·83	0·74, 0·94	502/6381	0·81	0·68, 0·98	0·86	0·66, 1·13	768/6381	0·77	0·66, 0·89	0·89	0·69, 1·14
T3	125 757	2647/6390	0·64	0·59, 0·69	0·77	0·69, 0·86	519/6390	0·63	0·53, 0·75	0·75	0·58, 0·97	1022/6390	0·79	0·68, 0·91	0·95	0·78, 1·16
* P* for trend			<0·0001			<0·0001		<0·0001			<0·05		<0·01		0·73	

*Model 1: Adjusted for age, sex and ethnicity. ^†^Model 2: Adjusted for age, sex, ethnicity, energy intake, BMI, PIR, diabetes, hypertension, alcohol consumption, saturated fatty acid intake, aspirin use, supplement use, CKD and history of CVD, excluding history of CVD for CVD death. Serum concentration ranges for each nutrient across tertiles: ^‡^T1(0–31·2), T2(31·2–55·6), T3(55·6–268·0) (µmol/l). ^§^T1(2·5–3·9), T2(3·9–4·2), T3(4·2–6·2) (mmol/l). ^||^T1(0–1·1), T2(1·1–1·7), T3(1·7–15·0) (µmol/l). ^¶^T1(0–0·04), T2(0·04–0·09), T3(0·09–3·76) (µmol/l). **T1(0–0·2), T2(0·2–0·4), T3(0·4–12·6) (µmol/l). ^††^T1(0–0·1), T2(0·1–0·2), T3(0·2–2·7) (µmol/l). ^‡‡^T1(0–0·3), T2(0·3–0·6), T3(0·6–2·9) (µmol/l). ^§§^T1(0–0·3), T2(0·3–0·4), T3(0·4–8·4) (µmol/l).

In a sensitivity analysis, additionally adjusting for 24-h intakes of fruit, vegetables, vitamin C, potassium, total dietary carotenoids and examination season, pooled hazard ratios changed only –1·5 % to +1·5 % for all-cause mortality, –3·6 % to +1·1 % for cancer mortality and –5·1 % to +1·2 % for CVD mortality (largest change for serum vitamin C), and all associations remained inverse and statistically significant. Multicollinearity diagnostics indicated no concerns (VIF ≤ 3·2; maximum condition index = 12·3), supporting retention of the pooled models as primary results.

When the associations between serum concentrations of vitamin C, potassium and carotenoids with all-cause mortality were stratified by follow-up period, no significant associations were observed in model 2 for the follow-up period of <5 years, except for *β*-carotene (online Supplementary Table 1). In the analysis using data from a follow-up period of 5 to less than 10 years, serum concentrations of total carotenoids and *β*-cryptoxanthin were associated with a reduced risk of all-cause mortality. Analyses of data from participants with follow-up periods of 15 years or more showed inverse associations between serum concentrations of total and individual carotenoids and all-cause mortality, while serum concentrations of vitamin C and potassium were not significantly associated with all-cause mortality.

Table 4 shows the sex-stratified HR and 5 % CI for all-cause, cancer and CVD mortality across serum concentrations of potassium, vitamin C and carotenoids. In these analyses, inverse associations between serum vitamin C concentrations and all-cause and cancer mortality were observed only in women. The inverse associations between serum concentrations of lycopene and *β*-carotene with cancer mortality, as well as between lutein and zeaxanthin concentrations and cancer mortality, were observed only among men.

**Table 4.** Sex-stratified multivariable HR and 95 % CI for all-cause, cancer and CVD mortality by tertiles of serum concentrations of vitamin C, potassium and total and individual carotenoids among US Adults aged ≥ 30 years participating in NHANES 1988–2006*

	Men										Women									
	Person-years	All-cause			Cancer			CVD			Person-years	All-cause			Cancer			CVD		
		*n* of death/ Total n	HR	95 % CI	*n* of death/ Total n	HR	95 % CI	*n* of death/ Total n	HR	95 % CI		*n* of death/ Total n	HR	95 % CI	*n* of death/ Total n	HR	95 % CI	*n* of death/ Total n	HR	95 % CI
Vitamin C^†^																				
T1	55 195	1398/2936	1·00		364/2936	1·00		473/2936	1·00		47 117	1383/2335	1·00		203/2335	1·00		322/2335	1·00	
T2	49 241	971/2593	0·95	0·81, 1·11	208/2593	0·81	0·59, 1·10	350/2593	1·02	0·72, 1·45	52 437	1723/2581	0·78	0·63, 0·97	162/2581	0·59	0·40, 0·89	305/2581	0·76	0·57, 1·02
T3	34 558	792/2092	1·06	0·89, 1·26	183/2092	1·02	0·74, 1·40	286/2092	0·94	0·68, 1·29	56 556	2009/3179	0·75	0·60, 0·92	187/3179	0·54	0·34, 0·85	461/3179	0·80	0·56, 1·15
* P*_for trend_			0·52			0·97			0·70				<0·01			<0·05			0·31	
Potassium^‡^																				
T1	43 948	860/2381	1·00		191/2381	1·00		325/2381	1·00		66 507	1104/3476	1·00		231/3476	1·00		378/3476	1·00	
T2	62 626	1191/3410	0·97	0·82, 1·14	297/3410	0·94	0·65, 1·37	402/3410	1·11	0·85, 1·44	68 596	1116/3543	0·98	0·84, 1·15	193/3543	0·87	0·57, 1·31	399/3543	1·06	0·80, 1·40
T3	60 144	1665/3569	1·08	0·95, 1·23	389/3569	1·04	0·75, 1·43	574/3569	1·18	0·92, 1·52	48 896	1250/2789	1·14	0·97, 1·35	208/2789	1·00	0·64, 1·57	459/2789	1·22	0·92, 1·61
* P*_for trend_			0·14			0·70			0·22				0·10			0·99			0·17	
Total carotenoids^§^																				
T1	56 296	1614/3283	1·00		412/3283	1·00		525/3283	1·00		58 086	1254/3106	1·00		238/3106	1·00		409/3106	1·00	
T2	57 538	1127/3106	0·72	0·62, 0·83	248/3106	0·65	0·49, 0·87	403/3106	0·84	0·61, 1·18	63 283	1075/3284	0·76	0·65, 0·90	208/3284	0·88	0·62, 1·25	401/3284	0·80	0·60, 1·06
T3	52 884	975/2971	0·68	0·59, 0·80	217/2971	0·53	0·36, 0·78	373/2971	0·93	0·66, 1·30	62 630	1141/3418	0·69	0·59, 0·80	186/3418	0·60	0·40, 0·90	426/3418	0·78	0·55, 1·11
* P*_for trend_			<0·0001			< 0·001			0·61				< 0·0001			< 0·05			0·18	
*α*-carotene^||^																				
T1	62 948	1543/3693	1·00		372/3693	1·00		510/3693	1·00		53 011	1051/2883	1·00		216/2883	1·00		353/2883	1·00	
T2	52 827	1147/2961	0·89	0·75, 1·05	271/2961	0·96	0·70, 1·30	433/2961	0·98	0·71, 1·35	56 671	1059/3076	0·80	0·67, 0·96	184/3076	1·01	0·68, 1·48	358/3076	0·58	0·45, 0·75
T3	50 943	1026/2706	0·79	0·67, 0·93	234/2706	0·78	0·56, 1·09	358/2706	0·86	0·60, 1·23	74 317	1360/3849	0·69	0·58, 0·82	232/3849	0·74	0·45, 1·20	525/3849	0·71	0·51, 0·98
* P*_for trend_			<0·01			0·14			0·40				< 0·001			0·17			0·17	
*β*-carotene^¶^																				
T1	67 878	1461/3799	1·00		361/3799	1·00		488/3799	1·00		50 288	834/2635	1·00		193/2635	1·00		256/2635	1·00	
T2	55 737	1158/3079	0·77	0·64, 0·93	273/3079	0·82	0·59, 1·14	409/3079	0·79	0·54, 1·16	61 382	1106/3232	0·74	0·62, 0·88	188/3232	0·88	0·56, 1·39	407/3232	0·77	0·55, 1·07
T3	43 104	1097/2482	0·74	0·63, 0·87	243/2482	0·68	0·48, 0·98	404/2482	0·81	0·58, 1·14	72 329	1530/3941	0·67	0·57, 0·80	251/3941	0·68	0·42, 1·10	573/3941	0·78	0·58, 1·05
* P*_for trend_			<0·001			<0·05			0·23				<0·0001			0·094			0·19	
*β*-cryptoxanthin**																				
T1	50 079	1438/3092	1·00		360/3092	1·00		481/3092	1·00		49 354	1064/2794	1·00		215/2794	1·00		365/2794	1·00	
T2	59 915	1279/3261	0·80	0·69, 0·93	303/3261	0·67	0·51, 0·87	452/3261	1·01	0·77, 1·33	66 136	1211/3431	0·90	0·77, 1·04	235/3431	1·07	0·77, 1·50	411/3431	0·67	0·51, 0·86
T3	56 724	999/3007	0·74	0·61, 0·91	214/3007	0·64	0·42, 0·98	368/3007	0·97	0·74, 1·27	68 509	1195/3583	0·71	0·59, 0·85	182/3583	0·64	0·42, 0·97	460/3583	0·69	0·49, 0·97
* P*_for trend_			<0·01			<0·05			0·85				<0·001			<0·05			0·055	
Lycopene^††^																				
T1	48 621	1726/2902	1·00		387/2902	1·00		619/2902	1·00		65 144	1765/3482	1·00		303/3482	1·00		639/3482	1·00	
T2	59 201	1186/3052	0·77	0·65, 0·91	289/3052	0·68	0·53, 0·88	392/3052	0·85	0·59, 1·21	67 485	1054/3349	0·89	0·77, 1·04	195/3349	0·73	0·50, 1·07	374/3349	0·91	0·71, 1·17
T3	58 897	804/3406	0·74	0·61, 0·88	201/3406	0·70	0·50, 0·99	290/3406	0·86	0·61, 1·23	51 370	651/2977	0·82	0·69, 0·98	134/2977	0·83	0·57, 1·22	223/2977	0·82	0·57, 1·19
* P*_for trend_			<0·001			<0·05			0·39				<0·05			0·28			0·27	
Lutein and zeaxanthin^‡‡^																				
T1	48 693	1206/3063	1·00		289/3063	1·00		398/3063	1·00		58 081	1074/3334	1·00		199/3334	1·00		349/3334	1·00	
T2	55 533	1191/3112	0·78	0·66, 0·93	297/3112	0·84	0·59, 1·18	400/3112	0·86	0·59, 1·24	62 653	1068/3269	0·91	0·78, 1·07	205/3269	0·89	0·59, 1·33	368/3269	0·96	0·72, 1·29
T3	62 492	1319/3185	0·72	0·61, 0·83	291/3185	0·67	0·48, 0·93	503/3185	0·89	0·64, 1·24	63 265	1328/3205	0·84	0·71, 0·99	228/3205	0·92	0·61, 1·41	519/3205	1·03	0·77, 1·39
* P*_for trend_			<0·0001			<0·05			0·51				<0·05			0·73			0·78	

*HR and 95 % CI were adjusted for age, ethnicity, energy intake, BMI, PIR, diabetes, hypertension, alcohol consumption, saturated fatty acid intake, aspirin use, supplement use, CKD and history of CVD, excluding history of CVD for CVD death. Serum concentration ranges for each nutrient across tertiles: ^†^T1(0–31·2), T2(31·2–55·6), T3(55·6–268·0) (µmol/l). ^‡^T1(2·5–3·9), T2(3·9–4·2), T3(4·2–6·2) (mmol/l). ^§^T1(0–1·1), T2(1·1–1·7), T3(1·7–15·0) (µmol/l). ^||^T1(0–0·04), T2(0·04–0·09), T3(0·09–3·76) (µmol/l). ^¶^T1(0–0·2), T2(0·2–0·4), T3(0·4–12·6) (µmol/l). **T1(0–0·1), T2(0·1–0·2), T3(0·2–2·7) (µmol/l). ^††^T1(0–0·3), T2(0·3–0·6), T3(0·6–2·9) (µmol/l). ^‡‡^T1(0–0·3), T2(0·3–0·4), T3(0·4–8·4) (µmol/l).

The multivariable HR and 95 % CI for all-cause, cancer and CVD mortality across serum concentrations of potassium, vitamin C and carotenoids were further stratified by BMI (online Supplementary Table 2) and smoking status (online Supplementary Table 3). The BMI and smoking-adjusted results were mostly consistent with the overall analysis. Specifically, when stratified by BMI, the inverse associations between serum vitamin C concentration and all-cause and cancer mortality, as well as the inverse association between serum *β*-cryptoxanthin, lycopene and lutein and zeaxanthin concentration and cancer mortality, were more pronounced in individuals with a BMI ≥ 25 kg/m². In contrast, the inverse associations between serum *β*-carotene concentration and cancer mortality, as well as serum lycopene concentration and CVD mortality, were more prominent in individuals with a BMI < 25 kg/m². When stratified by smoking status, the inverse associations between serum *α*-carotene, *β*-carotene and lycopene concentrations and cancer mortality were particularly notable among non-smokers. While the inverse association between serum vitamin C concentration and cancer mortality was significant among current smokers, there was a trend suggesting an increased risk of CVD mortality with higher serum potassium levels, although this was not statistically significant.

## Discussion

In this study, we aimed to examine the associations between serum concentrations of carotenoids, vitamin C and potassium, which are recognised as objective biomarkers of FV intake, and all-cause and cause-specific mortality, overcoming potential inaccuracies from self-reported dietary data. Before focusing on these associations, we first confirmed whether serum concentrations were significantly linked to actual dietary intakes of fruit, vegetables and nutrients. The analysis revealed that participants with higher serum concentrations of vitamin C and total carotenoids had significantly higher dietary intakes of fruits, vegetables, vitamin C, potassium and individual carotenoids compared with those with lower serum concentrations, which is consistent with previous findings^(37–39)^. Detailed US food sources for each carotenoid have been reported previously^(40)^.

While some studies^(41)^ have reported that higher serum vitamin C levels are associated with reduced mortality, others^(42)^ have found no significant association or have reported a U-shaped relationship, indicating an inconsistency. In our study, although the association between serum vitamin C and mortality was not significant in model 2 after adjusting for confounding factors, the subgroup analysis revealed that higher serum vitamin C levels were associated with a decrease in all-cause mortality and cancer mortality specifically among women. This disparity in findings between sexes may be a result of the greater overall mean of serum vitamin C among women participating in the NHANES cycles.

The results of this study indicated that although there were no clear significant differences between the highest and lowest serum potassium groups, there was a trend suggesting that higher serum potassium levels were associated with an increased risk of all-cause mortality. This finding was consistent with previous research^(43)^, which suggested that elevated serum potassium in CVD patients may increase the risk of cardiac arrhythmias, or that hyperkalaemia may occur in patients with impaired kidney function^(44)^. Additionally, elevated serum potassium may have contributed to inflammation and oxidative stress^(45)^.

Higher serum total carotenoid concentrations were associated with reduced all-cause mortality and cancer mortality, consistent with the findings of Pu et al.^(46)^. This association remained significant across sexes, BMI categories and participant smoking status. Regarding individual carotenoids, higher serum concentrations of *α*-carotene, *β*-carotene, *β*-cryptoxanthin, lycopene and lutein and zeaxanthin were significantly associated with reduced all-cause mortality. This finding aligns with the results reported by Hu et al.^(47)^ among CKD patients, as well as with the findings of Zhang et al^(36)^. In the studies by Zhu et al.^(34)^, who analysed hypertensive US adults from the NHANES III and NHANES 2001–2006, and by Lin et al.^(48)^, who focused on US adults with metabolic dysfunction-associated fatty liver disease from NHANES III, significant inverse associations were reported between serum *α*-carotene and lycopene and CVD mortality. However, in our study, no significant associations were found between serum *α*-carotene or lycopene and CVD mortality. These discrepancies could be attributed to differences in the study populations, the exclusion criteria applied and variations in the adjustment for confounding factors.

In the current study, *α*-carotene, *β*-carotene, *β*-cryptoxanthin, lycopene and lutein and zeaxanthin demonstrated a significantly inverse association with cancer mortality. These findings are consistent with previous research utilising NHANES III data^(49)^. Serum *β*-carotene was significantly associated with reduced all-cause mortality, cancer mortality and CVD mortality^(50,51)^. In the present study, higher levels of *β*-cryptoxanthin were significantly associated with reductions in all-cause mortality^(35)^ and cancer mortality. The potential cancer-preventive effects of *β*-cryptoxanthin may be attributed to its ability to enhance DNA repair and provide antioxidant protection against cellular damage^(52)^. Serum lycopene exhibited an inverse association with all-cause and cancer mortality, which is consistent with previous research using NHANES data on US adults with metabolic syndrome^(53)^, US adults with systemic lupus erythematosus^(54)^ and Japanese individuals aged 39 years and older who participated in the annual Comprehensive Health Examination Program^(55)^. The inverse association observed in this study between all-cause mortality and total carotenoids, *α*-carotene, *β*-carotene and lycopene is also in agreement with the results of Jayedi’s systematic review^(56)^.

Higher serum levels of lutein and zeaxanthin were significantly associated with reduced all-cause and cancer mortality, which is consistent with findings from previous studies^(57)^. While Goodman et al.^(58)^ reported that these inverse relationships were more pronounced among women than men, in our study, significant associations were observed only among men, individuals who are obese and smokers. In our study, each of these groups had lower mean concentrations of serum lutein and zeaxanthin. Therefore, they may have been more likely to experience a reduced risk of all-cause and cancer mortality with increased intake. In comparison, groups with higher average intakes may have already surpassed a potential maximum concentration threshold for these benefits.

The stronger associations observed with longer follow-up periods may be attributed to an increase in the number of mortality events, improving statistical power and allowing for more robust findings. In shorter follow-up periods, the reduced number of deaths likely limited the ability to detect significant relationships. Additionally, single measurements of serum biomarkers reflect more short-term intake and are subject to regression dilution over time, which may have weakened associations. However, longer follow-up periods may also exacerbate the effects of regression dilution, as the baseline biomarker levels may become less reflective of an individual’s long-term exposure, potentially weakening the observed associations. This suggests that while longer follow-up periods can increase statistical power, they may also introduce greater potential for measurement variability and dilution effects, though further research is needed to confirm this possibility.

This study has several strengths. First, we used a large, nationally representative sample of the US adult population, allowing for the investigation of the relationship between serum concentrations of vitamin C, potassium, total and individual carotenoids and mortality across participants with varying lifestyle and socio-demographic characteristics. Second, our study included a large number of deaths and a long-term follow-up of 18·8 years. Third, we utilised serum concentrations of carotenoids and vitamin C, which are more objective biomarkers, to assess their relationship with mortality. However, this study has several limitations. First, in this analysis, only a single measurement of serum concentrations of vitamin C, potassium and carotenoids from both NHANES III and NHANES 2001–2006 was utilised, without repeated measurements over time. Second, there may be competing risks between CVD and cancer mortality that could influence the results. Third, despite our efforts to harmonise variables between the 1988–1994 and 2001–2006 datasets, there may be systematic errors associated with combining these two datasets. Fourth, this study utilised only one-day 24-h dietary recalls data for both NHANES III and NHANES 2001–2006 to maintain consistency, despite the availability of 2-day 24-h dietary recalls data in NHANES 2001–2006. Fifth, although FV are the major sources of carotenoids, vitamin C and potassium^(40)^, these nutrients can also come from other dietary sources. Lastly, residual confounding factors may still exist in this analysis, even though we adjusted for a comprehensive set of relevant covariates available in the NHANES dataset. In particular, higher serum carotenoid concentrations may be correlated with other healthy behaviours, which could partially explain the observed associations despite multivariable adjustment.

### Conclusion

In conclusion, this study highlights the significant associations between serum levels of key nutrients found in FV such as carotenoids and vitamin C with all-cause, cancer and cardiovascular mortality in a nationally representative sample of US adults. These findings underscore the potential importance of these biomarkers in understanding mortality risk and the need for further research to elucidate the underlying mechanisms and implications for public health.

## Supporting information

Kim et al. supplementary material Kim et al. supplementary material

## Supplementary material

For supplementary material/s referred to in this article, please visit https://doi.org/10.1017/S000711452510531X

## References

[1] Aune D , Giovannucci E , Boffetta P , et al. (2017) Fruit and vegetable intake and the risk of cardiovascular disease, total cancer and all-cause mortality-a systematic review and dose-response meta-analysis of prospective studies. Int J Epidemiol 46, 1029–1056. 28338764 10.1093/ije/dyw319 PMC5837313

[2] Wang K , Chen Z , Shen M , et al. (2023) Dietary fruits and vegetables and risk of cardiovascular diseases in elderly Chinese. Eur J Public Health 33, 1088–1094. 37528047 10.1093/eurpub/ckad131 PMC10710356

[3] Madsen H , Sen A & Aune D (2023) Fruit and vegetable consumption and the risk of hypertension: a systematic review and meta-analysis of prospective studies. Eur J Nutr 62, 1941–1955. 37106252 10.1007/s00394-023-03145-5 PMC10349693

[4] Eggersdorfer M & Wyss A (2018) Carotenoids in human nutrition and health. Arch Biochem Biophys 652, 18–26. 29885291 10.1016/j.abb.2018.06.001

[5] Drewnowski A , Rock CL , Henderson SA , et al. (1997) Serum beta-carotene and vitamin C as biomarkers of vegetable and fruit intakes in a community-based sample of French adults. Am J Clin Nutr 65, 1796–1802. 9174475 10.1093/ajcn/65.6.1796

[6] Woodside JV , Draper J , Lloyd A , et al. (2017) Use of biomarkers to assess fruit and vegetable intake. Proc Nutr Soc 76, 308–315. 28347371 10.1017/S0029665117000325

[7] Saini RK , Nile SH & Park SW (2015) Carotenoids from fruits and vegetables: chemistry, analysis, occurrence, bioavailability and biological activities. Food Res Int 76, 735–750. 28455059 10.1016/j.foodres.2015.07.047

[8] Chew BP & Park JS (2004) Carotenoid action on the immune response. J Nutr 134, 257S–261S. 14704330 10.1093/jn/134.1.257S

[9] Ciccone MM , Cortese F , Gesualdo M , et al. (2013) Dietary intake of carotenoids and their antioxidant and anti-inflammatory effects in cardiovascular care. Mediators Inflamm 2013, 782137. 24489447 10.1155/2013/782137 PMC3893834

[10] Kesse-Guyot E , Andreeva VA , Ducros V , et al. (2014) Carotenoid-rich dietary patterns during midlife and subsequent cognitive function. Br J Nutr 111, 915–923. 24073964 10.1017/S0007114513003188

[11] Bungau S , Abdel-Daim MM , Tit DM , et al. (2019) Health benefits of polyphenols and carotenoids in age-related eye diseases. Oxid Med Cell Longev 2019, 9783429. 30891116 10.1155/2019/9783429 PMC6390265

[12] Rowles JL 3rd & Erdman JW Jr (2020) Carotenoids and their role in cancer prevention. Biochim Biophys Acta Mol Cell Biol Lipids 1865, 158613. 31935448 10.1016/j.bbalip.2020.158613

[13] Yao Y , Goh HM & Kim JE (2021) The roles of carotenoid consumption and bioavailability in cardiovascular health. Antioxidants (Basel) 10, 1978. 34943081 10.3390/antiox10121978 PMC8750451

[14] Chambial S , Dwivedi S , Shukla KK , et al. (2013) Vitamin C in disease prevention and cure: an overview. Indian J Clin Biochem 28, 314–328. 24426232 10.1007/s12291-013-0375-3 PMC3783921

[15] Padayatty SJ , Sun H , Wang Y , et al. (2004) Vitamin C pharmacokinetics: implications for oral and intravenous use. Ann Intern Med 140, 533–537. 15068981 10.7326/0003-4819-140-7-200404060-00010

[16] He FJ & MacGregor GA (2008) Beneficial effects of potassium on human health. Physiol Plant 133, 725–735. 18724413 10.1111/j.1399-3054.2007.01033.x

[17] Weaver CM (2013) Potassium and health. Adv Nutr 4, 368S–377S. 23674806 10.3945/an.112.003533 PMC3650509

[18] McDonough AA & Youn JH (2017) Potassium homeostasis: the knowns, the unknowns, and the health benefits. Physiology (Bethesda) 32, 100–111. 28202621 10.1152/physiol.00022.2016 PMC5337831

[19] U.S. Department of Health and Human Services *National Health and Nutrition Examination Survey III, 1988–1994*. Hyattsville, MD: Centers for Disease Control and Prevention (CDC). (accessed January 2025).

[20] U.S. Department of Health and Human Services *National Health and Nutrition Examination Survey Data 2001–2002*. Hyattsville, MD: Centers for Disease Control and Prevention (CDC). (accessed January 2025).

[21] U.S. Department of Health and Human Services *National Health and Nutrition Examination Survey Data 2003–2004*. Hyattsville, MD: Centers for Disease Control and Prevention (CDC). (accessed January 2025).

[22] U.S. Department of Health and Human Services *National Health and Nutrition Examination Survey Data 2005–2006*. Hyattsville, MD: Centers for Disease Control and Prevention (CDC). (accessed January 2025).

[23] Curtin SC , Tejada-Vera B & Bastian BA (2024) *Deaths: Leading Causes for 2021. National Vital Statistics Reports*, vol. 73. Hyattsville, MD: National Center for Health Statistics, Centers for Disease Control and Prevention.

[24] U.S. Department of Agriculture ARS (2004) *USDA Food and Nutrient Database for Dietary Studies*, 1.0. Beltsville, MD: Agricultural Research Service, Food Surveys Research Group.

[25] U.S. Department of Agriculture ARS (2006) *USDA Food and Nutrient Database for Dietary Studies*, 2.0. Beltsville, MD: Agricultural Research Service, Food Surveys Research Group.

[26] U.S. Department of Agriculture ARS (2008) *USDA Food and Nutrient Database for Dietary Studies*, 3.0. Beltsville, MD: Agricultural Research Service, Food Surveys Research Group.

[27] Gunter EW , Lewis BG & Koncikowski SM (1996) *Laboratory Procedures Used for the Third National Health and Nutrition Examination Survey (NHANES III), 1988–1994*. Atlanta, GA: Department of Health and Human Services, Centers for Disease Control and Prevention.

[28] National Center for Health Statistics, NHANES (2006) *Laboratory/Medical Technologists Procedures Manual (LPM)*. Hyattsville, MD: Department of Health and Human Services, Centers for Disease Control and Prevention.

[29] National Center for Health Statistics (2022) *2019 Public-Use Linked Mortality Files*. Hyattsville, MD: National Center for Health Statistics.

[30] World Health Organization *International Statistical Classification of Diseases and Related Health Problems*, 9th revision. Geneva: World Health Organization. (accessed January 2025).

[31] World Health Organization (2019) *International Statistical Classification of Diseases and Related Health Problems*, 10th revision. Geneva: World Health Organization. (accessed January 2025).

[32] Ronksley PE , Brien SE , Turner BJ , et al. (2011) Association of alcohol consumption with selected cardiovascular disease outcomes: a systematic review and meta-analysis. BMJ 342, d671. 21343207 10.1136/bmj.d671 PMC3043109

[33] Inker LA , Eneanya ND , Coresh J , et al. (2021) New creatinine- and cystatin C-based equations to estimate GFR without race. N Engl J Med 385, 1737–1749. 34554658 10.1056/NEJMoa2102953 PMC8822996

[34] Zhu X , Cheang I , Tang Y , et al. (2023) Associations of serum carotenoids with risk of all-cause and cardiovascular mortality in hypertensive adults. J Am Heart Assoc 12, e027568. 36752230 10.1161/JAHA.122.027568 PMC10111495

[35] De Waart FG , Schouten EG , Stalenhoef AF , et al. (2001) Serum carotenoids, *α*-tocopherol and mortality risk in a prospective study among Dutch elderly. Int J Epidemiol 30, 136–143. 11171874 10.1093/ije/30.1.136

[36] Zhang G , Li X & Zheng X (2024) Associations of serum carotenoids with asthma and mortality in the US adults. Heliyon 10, e24992. 38318021 10.1016/j.heliyon.2024.e24992 PMC10840010

[37] Dehghan M , Akhtar-Danesh N , McMillan CR , et al. (2007) Is plasma vitamin C an appropriate biomarker of vitamin C intake? A systematic review and meta-analysis. Nutr J 6, 41. 17997863 10.1186/1475-2891-6-41 PMC2200644

[38] Dauchet L , Peneau S , Bertrais S , et al. (2008) Relationships between different types of fruit and vegetable consumption and serum concentrations of antioxidant vitamins. Br J Nutr 100, 633–641. 18279554 10.1017/S000711450892170X

[39] Campbell DR , Gross MD , Martini MC , et al. (1994) Plasma carotenoids as biomarkers of vegetable and fruit intake. Cancer Epidemiol Biomarkers Prev 3, 493–500. 8000300

[40] Kim K , Madore MP & Chun OK (2023) Changes in intake and major food sources of carotenoids among U.S. adults between 2009–2018. Metabolites 14, 13. 38248816 10.3390/metabo14010013 PMC10820268

[41] Aune D , Keum N , Giovannucci E , et al. (2018) Dietary intake and blood concentrations of antioxidants and the risk of cardiovascular disease, total cancer, and all-cause mortality: a systematic review and dose-response meta-analysis of prospective studies. Am J Clin Nutr 108, 1069–1091. 30475962 10.1093/ajcn/nqy097 PMC6250988

[42] Tian T , Shao J , Shen Z , et al. (2022) Association of serum vitamin C with all-cause and cause-specific death: data from National Health and Nutrition Examination Survey (NHANES 2003–2006). Nutrition 101, 111696. 35660506 10.1016/j.nut.2022.111696

[43] Hughes-Austin JM , Rifkin DE , Beben T , et al. (2017) The relation of serum potassium concentration with cardiovascular events and mortality in community-living individuals. Clin J Am Soc Nephrol 12, 245–252. 28143865 10.2215/CJN.06290616 PMC5293337

[44] Hoppe LK , Muhlack DC , Koenig W , et al. (2018) Association of abnormal serum potassium levels with arrhythmias and cardiovascular mortality: a systematic review and meta-analysis of observational studies. Cardiovasc Drugs Ther 32, 197–212. 29679302 10.1007/s10557-018-6783-0

[45] Udensi UK & Tchounwou PB (2017) Potassium homeostasis, oxidative stress, and human disease. Int J Clin Exp Physiol 4, 111–122. 29218312 10.4103/ijcep.ijcep_43_17 PMC5716641

[46] Pu L , Zhang R , Wang X , et al. (2022) Associations of serum biomarkers of fruit and vegetable intake with the risk of cause-specific mortality and all-cause mortality: a national prospective cohort study. Front Nutr 9, 874943. 35634408 10.3389/fnut.2022.874943 PMC9134271

[47] Hu Y , Cai X , Zhang N , et al. (2022) Relation between dietary carotenoid intake, serum concentration, and mortality risk of CKD patients among US adults: national Health and Nutrition Examination Survey 2001–2014. Front Med (Lausanne) 9, 871767. 35872751 10.3389/fmed.2022.871767 PMC9304649

[48] Lin B , Liu Z , Li D , et al. (2024) Associations of serum carotenoids with all-cause and cardiovascular mortality in adults with MAFLD. Nutr Metab Cardiovasc Dis 34, 2315–2324. 39003130 10.1016/j.numecd.2024.06.001

[49] Min KB & Min JY (2014) Serum carotenoid levels and risk of lung cancer death in US adults. Cancer Sci 105, 736–743. 24673770 10.1111/cas.12405 PMC4317899

[50] Qiu Z , Chen X , Geng T , et al. (2022) Associations of serum carotenoids with risk of cardiovascular mortality among individuals with type 2 diabetes: results from NHANES. Diabetes Care 45, 1453–1461. 35503926 10.2337/dc21-2371

[51] Ito Y , Suzuki S , Yagyu K , et al. (1997) Relationship between serum carotenoid levels and cancer death rates in the residents, living in a rural area of Hokkaido, Japan. J Epidemiol 7, 1–8. 9127566 10.2188/jea.7.1

[52] Lorenzo Y , Azqueta A , Luna L , et al. (2009) The carotenoid beta-cryptoxanthin stimulates the repair of DNA oxidation damage in addition to acting as an antioxidant in human cells. Carcinog 30, 308–314. 10.1093/carcin/bgn270 19056931

[53] Han GM , Meza JL , Soliman GA , et al. (2016) Higher levels of serum lycopene are associated with reduced mortality in individuals with metabolic syndrome. Nutr Res 36, 402–407. 27101758 10.1016/j.nutres.2016.01.003

[54] Han GM & Han XF (2016) Lycopene reduces mortality in people with systemic lupus erythematosus: a pilot study based on the third national health and nutrition examination survey. J Dermatolog Treat 27, 430–435. 26762689 10.3109/09546634.2015.1133879

[55] Ito Y , Suzuki K , Suzuki S , et al. (2002) Serum antioxidants and subsequent mortality rates of all causes or cancer among rural Japanese inhabitants. Int J Vitam Nutr Res 72, 237–250. 12214561 10.1024/0300-9831.72.4.237

[56] Jayedi A , Rashidy-Pour A , Parohan M , et al. (2018) Dietary antioxidants, circulating antioxidant concentrations, total antioxidant capacity, and risk of all-cause mortality: a systematic review and dose-response meta-analysis of prospective observational studies. Adv Nutr 9, 701–716. 30239557 10.1093/advances/nmy040 PMC6247336

[57] Sato R , Helzlsouer KJ , Alberg AJ , et al. (2002) Prospective study of carotenoids, tocopherols, and retinoid concentrations and the risk of breast cancer. Cancer Epidemiol Biomarkers Prev 11, 451–457. 12010859

[58] Goodman GE , Schaffer S , Omenn GS , et al. (2003) The association between lung and prostate cancer risk, and serum micronutrients: results and lessons learned from beta-carotene and retinol efficacy trial. Cancer Epidemiol Biomarkers Prev 12, 518–526. 12814997

