# Supplementary material for: Associations between serum biomarkers of fruit and vegetable intake and all-cause, cancer and CVD mortality among US adults
Source: Br J Nutr. 2025 Oct 3;134(10):836–49. doi: 10.1017/S000711452510531X (PMC12795639; doi:10.1017/S000711452510531X)
Supplement: Kim et al. supplementary material [file S000711452510531Xsup001.docx]

Supplementary Table 1. Baseline follow-up period-stratified HRs and 95% CIs for all-cause mortality by tertiles of serum concentrations of vitamin C, potassium, and carotenoids among U.S. adults aged ≥ 30 years participating in NHANES 1988-2006

|  | Person-years <5 years | | | 5≤Person-years <10 | | | 10≤Person-years <15 | | | Person-years≥15 | | |  |
| --- | --- | --- | --- | --- | --- | --- | --- | --- | --- | --- | --- | --- | --- |
|  | N of death/n | Model 1^*^ | Model 2^†^ | N of death/n | Model 1^*^ | Model 2^†^ | N of death/n | Model 1^*^ | Model 2^†^ | N of death/n | Model 1^*^ | Model 2^†^ | |
| Vitamin C |  |  |  |  |  |  |  |  |  |  |  |  | |
| T1 | 467/5360 | 1.0 | 1.0 | 524/4893 | 1.0 | 1.0 | 554/4369 | 1.0 | 1.0 | 929/3815 | 1.0 | 1.0 | |
| T2 | 316/5351 | 0.70 (0.56-0.87) | 0.94 (0.67-1.32) | 441/5035 | 0.76 (0.62-0.93) | 0.98 (0.69-1.39) | 449/4594 | 0.60 (0.51-0.72) | 0.81 (0.62-1.05) | 765/4145 | 0.71 (0.62-0.81) | 0.88 (0.73-1.06) | |
| T3 | 388/5379 | 0.55 (0.44-0.67) | 0.87 (0.59-1.26) | 513/4991 | 0.58 (0.49-0.69) | 0.97 (0.70-1.36) | 516/4478 | 0.47 (0.39-0.56) | 0.64 (0.47-0.88) | 653/3962 | 0.74 (0.63-0.88) | 1.04 (0.85-1.29) | |
| P for trend |  | <0.0001 | 0.45 |  | <0.0001 | 0.88 |  | <0.0001 | <0.01 |  | <0.001 | 0.67 | |
| Potassium |  |  |  |  |  |  |  |  |  |  |  |  | |
| T1 | 352/5967 | 1.0 | 1.0 | 444/5615 | 1.0 | 1.0 | 480/5171 | 1.0 | 1.0 | 798/4691 | 1.0 | 1.0 | |
| T2 | 383/7063 | 0.88 (0.71-1.08) | 0.82 (0.54-1.23) | 527/6680 | 0.94 (0.80-1.10) | 0.95 (0.72-1.26) | 604/6153 | 0.91 (0.78-1.07) | 1.04 (0.81-1.33) | 903/5549 | 1.00 (0.84-1.18) | 1.01 (0.84-1.22) | |
| T3 | 668/6572 | 1.10 (0.91-1.34) | 0.93 (0.66-1.30) | 833/5904 | 1.19 (1.02-1.39) | 1.10 (0.82-1.48) | 744/5071 | 1.15 (0.95-1.38) | 1.25 (0.94-1.68) | 884/4327 | 1.12 (0.97-1.30) | 1.12 (0.96-1.32) | |
| P for trend |  | 0.17 | 0.83 |  | <0.05 | 0.39 |  | 0.094 | 0.11 |  | 0.093 | 0.12 | |
| Total carotenoids |  |  |  |  |  |  |  |  |  |  |  |  | |
| T1 | 647/6524 | 1.0 | 1.0 | 727/5877 | 1.0 | 1.0 | 688/5150 | 1.0 | 1.0 | 992/4462 | 1.0 | 1.0 | |
| T2 | 371/6540 | 0.60 (0.50-0.73) | 0.89 (0.62-1.27) | 555/6169 | 0.67 (0.57-0.78) | 0.76 (0.60-0.96) | 591/5614 | 0.69 (0.60-0.81) | 0.82 (0.64-1.06) | 827/5023 | 0.63 (0.55-0.73) | 0.66 (0.55-0.79) | |
| T3 | 385/6538 | 0.54 (0.45-0.65) | 0.75 (0.53-1.06) | 522/6153 | 0.52 (0.45-0.61) | 0.77 (0.59-1.00) | 549/5631 | 0.52 (0.44-0.60) | 0.83 (0.66-1.04) | 766/5082 | 0.49 (0.42-0.57) | 0.58 (0.49-0.70) | |
| P for trend |  | <0.0001 | 0.10 |  | <0.0001 | <0.05 |  | <0.0001 | 0.10 |  | <0.0001 | <0.0001 | |
| α-carotene |  |  |  |  |  |  |  |  |  |  |  |  | |
| T1 | 581/6763 | 1.0 | 1.0 | 654/6182 | 1.0 | 1.0 | 695/5528 | 1.0 | 1.0 | 851/4833 | 1.0 | 1.0 | |
| T2 | 414/6170 | 0.56 (0.47-0.67) | 0.78 (0.55-1.11) | 585/5756 | 0.73 (0.63-0.84) | 0.91 (0.72-1.14) | 537/5171 | 0.57 (0.49-0.67) | 0.77 (0.60-0.99) | 803/4634 | 0.77 (0.64-0.91) | 0.90 (0.73-1.11) | |
| T3 | 408/6669 | 0.50 (0.41-0.60) | 0.73 (0.52-1.03) | 565/6261 | 0.53 (0.45-0.62) | 0.80 (0.61-1.06) | 596/5696 | 0.48 (0.42-0.55) | 0.72 (0.58-0.90) | 931/5100 | 0.53 (0.45-0.63) | 0.74 (0.59-0.92) | |
| P for trend |  | <0.0001 | 0.077 |  | <0.0001 | 0.12 |  | <0.0001 | <0.01 |  | <0.0001 | <0.01 | |
| β-carotene |  |  |  |  |  |  |  |  |  |  |  |  | |
| T1 | 451/6594 | 1.0 | 1.0 | 556/6143 | 1.0 | 1.0 | 589/5587 | 1.0 | 1.0 | 859/4998 | 1.0 | 1.0 | |
| T2 | 457/6457 | 0.69 (0.55-0.85) | 0.84 (0.60-1.17) | 563/6000 | 0.64 (0.55-0.75) | 0.83 (0.62-1.11) | 576/5437 | 0.68 (0.58-0.79) | 0.87 (0.65-1.16) | 814/4861 | 0.65 (0.55-0.76) | 0.69 (0.55-0.85) | |
| T3 | 495/6551 | 0.55 (0.46-0.66) | 0.70 (0.49-0.99) | 685/6056 | 0.57 (0.49-0.65) | 0.76 (0.58-1.01) | 663/5371 | 0.51 (0.44-0.59) | 0.76 (0.58-0.99) | 912/4708 | 0.53 (0.45-0.63) | 0.66 (0.53-0.82) | |
| P for trend |  | <0.0001 | <0.05 |  | <0.0001 | 0.064 |  | <0.0001 | <0.05 |  | <0.0001 | <0.001 | |
| β-cryptoxanthin |  |  |  |  |  |  |  |  |  |  |  |  | |
| T1 | 567/6077 | 1.0 | 1.0 | 663/5510 | 1.0 | 1.0 | 679/4847 | 1.0 | 1.0 | 784/4168 | 1.0 | 1.0 | |
| T2 | 483/6840 | 0.83 (0.68-1.01) | 1.21 (0.86-1.70) | 601/6357 | 0.75 (0.66-0.86) | 0.89 (0.73-1.10) | 605/5756 | 0.68 (0.60-0.78) | 0.84 (0.67-1.06) | 949/5151 | 0.69 (0.59-0.81) | 0.75 (0.64-0.89) | |
| T3 | 353/6685 | 0.59 (0.49-0.72) | 0.82 (0.54-1.25) | 540/6332 | 0.56 (0.49-0.65) | 0.75 (0.57-0.99) | 544/5792 | 0.51 (0.44-0.60) | 0.81 (0.62-1.04) | 852/5248 | 0.50 (0.42-0.59) | 0.64 (0.52-0.78) | |
| P for trend |  | <0.0001 | 0.42 |  | <0.0001 | <0.05 |  | <0.0001 | 0.095 |  | <0.0001 | <0.0001 | |
| Lycopene |  |  |  |  |  |  |  |  |  |  |  |  | |
| T1 | 773/6537 | 1.0 | 1.0 | 894/5764 | 1.0 | 1.0 | 819/4870 | 1.0 | 1.0 | 1229/4051 | 1.0 | 1.0 | |
| T2 | 358/6594 | 0.61 (0.51-0.74) | 0.77 (0.57-1.05) | 538/6236 | 0.77 (0.65-0.90) | 0.81 (0.62-1.04) | 553/5698 | 0.78 (0.67-0.90) | 0.86 (0.69-1.08) | 926/5145 | 0.78 (0.68-0.91) | 0.84 (0.72-0.97) | |
| T3 | 272/6471 | 0.60 (0.49-0.73) | 0.75 (0.52-1.09) | 372/6199 | 0.58 (0.48-0.70) | 0.87 (0.63-1.20) | 456/5827 | 0.70 (0.60-0.82) | 0.90 (0.72-1.14) | 430/5371 | 0.66 (0.55-0.79) | 0.70 (0.58-0.84) | |
| P for trend |  | <0.0001 | 0.092 |  | <0.0001 | 0.31 |  | <0.0001 | 0.32 |  | <0.0001 | <0.001 | |
| Lutein & zeaxanthin |  |  |  |  |  |  |  |  |  |  |  |  | |
| T1 | 525/6535 | 1.0 | 1.0 | 646/6010 | 1.0 | 1.0 | 637/5364 | 1.0 | 1.0 | 636/4727 | 1.0 | 1.0 | |
| T2 | 412/6527 | 0.64 (0.54-0.76) | 0.77 (0.58-1.04) | 590/6115 | 0.76 (0.65-0.89) | 0.78 (0.58-1.05) | 562/5525 | 0.74 (0.64-0.86) | 0.91 (0.72-1.14) | 815/4963 | 0.73 (0.62-0.87) | 0.82 (0.68-0.99) | |
| T3 | 466/6540 | 0.81 (0.68-0.97) | 1.04 (0.75-1.42) | 568/6074 | 0.63 (0.53-0.74) | 0.77 (0.59-1.01) | 629/5506 | 0.63 (0.54-0.73) | 0.82 (0.64-1.05) | 1134/4877 | 0.59 (0.50-0.70) | 0.68 (0.55-0.83) | |
| P for trend |  | <0.05 | 0.75 |  | <0.0001 | 0.067 |  | <0.0001 | 0.12 |  | <0.0001 | <0.001 | |

^*^ Model1: Adjusted for age, sex, and ethnicity. ^†^ Model2: Adjusted for age, sex, ethnicity, energy intake, BMI, PIR, diabetes, hypertension, alcohol consumption, saturated fatty acid intake, aspirin use, supplement use, CKD and history of CVD; excluding history of CVD for CVD death.

Supplementary Table 2. Baseline BMI-stratified HRs and 95% CIs for all-cause, cancer, and CVD mortality by tertiles of serum concentrations of vitamin C, potassium, and carotenoids among U.S. adults aged ≥ 30 years participating in NHANES 1988-2006^*^

|  | BMI < 25 kg/m^2^ | | | | | | | BMI ≥ 25 kg/m^2^ | | | | | | | | |
| --- | --- | --- | --- | --- | --- | --- | --- | --- | --- | --- | --- | --- | --- | --- | --- | --- |
|  | Person-years | All-cause mortality | | Cancer mortality | | CVD mortality | | Person-years | All-cause mortality | | | Cancer mortality | | CVD mortality | |  |
|  |  | N of death/n | HR  (95% CI) | N of death/n | HR  (95% CI) | N of death/n | HR  (95% CI) |  | N of death/n | HR  (95% CI) | N of death/n | | HR  (95% CI) | N of death/n | HR  (95% CI) |  |
| Vitamin C |  |  |  |  |  |  |  |  |  |  |  | |  |  |  |  |
| T1 | 30,889 | 747/1602 | 1.00 | 209/1602 | 1.00 | 219/1602 | 1.00 | 71,030 | 1584/3634 | 1.00 | 357/3634 | | 1.00 | 570/3634 | 1.00 |  |
| T2 | 28,773 | 469/1385 | 0.95 (0.75-1.22) | 88/1385 | 0.68 (0.44-1.06) | 155/1385 | 1.09 (0.68-1.74) | 72,605 | 1342/3762 | 0.86 (0.74-0.99) | 282/3762 | | 0.72 (0.50-1.02) | 496/3762 | 0.86 (0.64-1.16) |  |
| T3 | 34,091 | 726/1920 | 1.06 (0.86-1.30) | 126/1920 | 0.82 (0.56-1.21) | 280/1920 | 1.08 (0.69-1.69) | 56,593 | 1221/3316 | 0.84 (0.71-0.99) | 243/3316 | | 0.70 (0.49-0.99) | 461/3316 | 0.85 (0.61-1.17) |  |
| P for trend |  |  | 0.53 |  | 0.36 |  | 0.76 |  |  | <0.05 |  | | <0.05 |  | 0.32 |  |
| Potassium |  |  |  |  |  |  |  |  |  |  |  | |  |  |  |  |
| T1 | 34,636 | 562/1769 | 1.00 | 120/1769 | 1.00 | 179/1769 | 1.00 | 74,974 | 1373/4023 | 1.00 | 299/4023 | | 1.00 | 518/4023 | 1.00 |  |
| T2 | 39,056 | 715/2058 | 1.14 (0.91-1.44) | 160/2058 | 1.10 (0.67-1.82) | 235/2058 | 1.59 (1.07-2.36) | 91,399 | 1555/4829 | 0.89 (0.77-1.04) | 325/4829 | | 0.79 (0.58-1.07) | 554/4829 | 0.93 (0.72-1.21) |  |
| T3 | 35,948 | 948/2061 | 1.11 (0.92-1.33) | 191/2061 | 1.02 (0.64-1.62) | 324/2061 | 1.51 (1.02-2.23) | 72,271 | 1889/4203 | 1.10 (0.97-1.26) | 402/4203 | | 0.99 (0.70-1.38) | 675/4203 | 1.11 (0.90-1.38) |  |
| P for trend |  |  | 0.36 |  | 0.99 |  | 0.065 |  |  | 0.053 |  | | 0.90 |  | 0.21 |  |
| Total carotenoids |  |  |  |  |  |  |  |  |  |  |  | |  |  |  |  |
| T1 | 29,858 | 837/1701 | 1.00 | 205/1701 | 1.00 | 259/1701 | 1.00 | 83,239 | 1952/4569 | 1.00 | 440/4569 | | 1.00 | 647/4569 | 1.00 |  |
| T2 | 36,930 | 629/1872 | 0.74 (0.60-0.90) | 136/1872 | 0.82 (0.58-1.17) | 199/1872 | 0.75 (0.53-1.06) | 83,210 | 1535/4455 | 0.74 (0.65-0.85) | 316/4455 | | 0.68 (0.52-0.88) | 591/4455 | 0.88 (0.68-1.14) |  |
| T3 | 42,852 | 759/2315 | 0.66 (0.54-0.81) | 130/2315 | 0.54 (0.33-0.89) | 280/2315 | 0.76 (0.53-1.09) | 72,195 | 1330/4031 | 0.70 (0.60-0.82) | 270/4031 | | 0.54 (0.38-0.76) | 509/4031 | 0.93 (0.72-1.20) |  |
| P for trend |  |  | <0.001 |  | <0.05 |  | 0.17 |  |  | <0.0001 |  | | <0.001 |  | 0.52 |  |
| α-carotene |  |  |  |  |  |  |  |  |  |  |  | |  |  |  |  |
| T1 | 30,446 | 752/1715 | 1.00 | 202/1715 | 1.00 | 211/1715 | 1.00 | 84,379 | 1764/4749 | 1.00 | 382/4749 | | 1.00 | 624/4749 | 1.00 |  |
| T2 | 30,408 | 588/1644 | 0.81 (0.62-1.08) | 113/1644 | 0.97 (0.62-1.53) | 220/1644 | 0.78 (0.51-1.20) | 78,403 | 1579/4330 | 0.88 (0.75-1.03) | 336/4330 | | 0.97 (0.67-1.39) | 559/4330 | 0.83 (0.64-1.07) |  |
| T3 | 48,786 | 885/2529 | 0.75 (0.60-0.93) | 156/2529 | 0.75 (0.48-1.18) | 307/2529 | 0.76 (0.49-1.19) | 75,862 | 1474/3976 | 0.75 (0.63-0.89) | 308/3976 | | 0.74 (0.50-1.10) | 564/3976 | 0.88 (0.67-1.15) |  |
| P for trend |  |  | <0.01 |  | 0.18 |  | 0.27 |  |  | <0.01 |  | | 0.13 |  | 0.38 |  |
| β-carotene |  |  |  |  |  |  |  |  |  |  |  | |  |  |  |  |
| T1 | 28,401 | 650/1554 | 1.00 | 179/1554 | 1.00 | 185/1554 | 1.00 | 88,733 | 1592/4793 | 1.00 | 372/4793 | | 1.00 | 541/4793 | 1.00 |  |
| T2 | 34,398 | 576/1773 | 0.69 (0.55-0.88) | 125/1773 | 0.66 (0.46-0.96) | 186/1773 | 0.78 (0.53-1.14) | 81,982 | 1642/4466 | 0.81 (0.69-0.95) | 333/4466 | | 0.96 (0.69-1.33) | 615/4466 | 0.81 (0.61-1.08) |  |
| T3 | 46,840 | 999/2561 | 0.63 (0.51-0.80) | 167/2561 | 0.50 (0.34-0.74) | 367/2561 | 0.79 (0.55-1.12) | 67,929 | 1583/3796 | 0.75 (0.65-0.86) | 321/3796 | | 0.78 (0.56-1.08) | 591/3796 | 0.82 (0.64-1.04) |  |
| P for trend |  |  | <0.001 |  | <0.001 |  | 0.24 |  |  | <0.001 |  | | 0.14 |  | 0.11 |  |
| β-cryptoxanthin |  |  |  |  |  |  |  |  |  |  |  | |  |  |  |  |
| T1 | 28,421 | 778/1678 | 1.00 | 189/1678 | 1.00 | 242/1678 | 1.00 | 69,805 | 1650/4095 | 1.00 | 380/4095 | | 1.00 | 582/4095 | 1.00 |  |
| T2 | 38,107 | 726/1974 | 0.88 (0.68-1.15) | 163/1974 | 1.01 (0.67-1.54) | 228/1974 | 0.73 (0.48-1.12) | 87,225 | 1719/4649 | 0.83 (0.73-0.95) | 371/4649 | | 0.71 (0.55-0.93) | 616/4649 | 0.90 (0.72-1.11) |  |
| T3 | 43,111 | 721/2236 | 0.74 (0.57-0.95) | 119/2236 | 0.64 (0.37-1.10) | 268/2236 | 0.82 (0.58-1.17) | 81,614 | 1448/4311 | 0.71 (0.60-0.84) | 275/4311 | | 0.59 (0.42-0.83) | 549/4311 | 0.83 (0.63-1.10) |  |
| P for trend |  |  | <0.05 |  | 0.098 |  | 0.34 |  |  | <0.0001 |  | | <0.01 |  | 0.18 |  |
| Lycopene |  |  |  |  |  |  |  |  |  |  |  | |  |  |  |  |
| T1 | 34,298 | 1108/1975 | 1.00 | 217/1975 | 1.00 | 393/1975 | 1.00 | 78,426 | 2307/4306 | 1.00 | 469/4306 | | 1.00 | 834/4306 | 1.00 |  |
| T2 | 41,303 | 697/2011 | 0.80 (0.66-0.97) | 151/2011 | 0.65 (0.42-1.03) | 213/2011 | 0.73 (0.55-0.97) | 84,575 | 1506/4321 | 0.85 (0.74-0.98) | 327/4321 | | 0.73 (0.55-0.97) | 544/4321 | 0.98 (0.73-1.32) |  |
| T3 | 34,039 | 420/1902 | 0.80 (0.63-1.00) | 103/1902 | 0.86 (0.52-1.44) | 132/1902 | 0.67 (0.44-1.03) | 75,644 | 1004/4428 | 0.77 (0.65-0.92) | 230/4428 | | 0.68 (0.50-0.93) | 369/4428 | 0.94 (0.75-1.19) |  |
| P for trend |  |  | <0.05 |  | 0.48 |  | <0.05 |  |  | <0.01 |  | | <0.05 |  | 0.63 |  |
| Lutein & zeaxanthin |  |  |  |  |  |  |  |  |  |  |  | |  |  |  |  |
| T1 | 28,585 | 644/1688 | 1.00 | 142/1688 | 1.00 | 199/1688 | 1.00 | 76,693 | 1550/4573 | 1.00 | 340/4573 | | 1.00 | 520/4573 | 1.00 |  |
| T2 | 37,203 | 711/1988 | 0.84 (0.69-1.02) | 159/1988 | 0.80 (0.50-1.29) | 225/1988 | 0.84 (0.58-1.21) | 80,322 | 1513/4333 | 0.84 (0.72-0.97) | 339/4333 | | 0.90 (0.65-1.24) | 529/4333 | 0.88 (0.66-1.19) |  |
| T3 | 43,852 | 870/2212 | 0.80 (0.65-0.99) | 170/2212 | 1.00 (0.63-1.56) | 314/2212 | 0.85 (0.60-1.20) | 81,629 | 1754/4149 | 0.76 (0.66-0.87) | 347/4149 | | 0.63 (0.46-0.85) | 698/4149 | 0.97 (0.76-1.24) |  |
| P for trend |  |  | <0.05 |  | 0.91 |  | 0.43 |  |  | <0.001 |  | | <0.01 |  | 0.89 |  |

^*^ HRs and 95% CIs were adjusted for age, ethnicity, energy intake, BMI, PIR, diabetes, hypertension, alcohol consumption, saturated fatty acid intake, aspirin use, supplement use, CKD and history of CVD; excluding history of CVD for CVD death.

Supplementary Table 3. Baseline smoking status-stratified HRs and 95% CIs for all-cause, cancer, and CVD mortality by tertiles of serum concentrations of vitamin C, potassium, and carotenoids among U.S. adults aged ≥ 30 years participating in NHANES 1988-2006^*^

|  | Current smokers | | | | | | | Non-smokers | | | | | | |
| --- | --- | --- | --- | --- | --- | --- | --- | --- | --- | --- | --- | --- | --- | --- |
|  | Person-years | All-cause mortality | | Cancer mortality | | CVD mortality | | Person-years | All-cause mortality | | Cancer mortality | | CVD mortality | |
|  |  | N of death/n | HR  (95% CI) | N of death/n | HR  (95% CI) | N of death/n | HR  (95% CI) |  | N of death/n | HR  (95% CI) | N of death/n | HR  (95% CI) | N of death/n | HR  (95% CI) |
| Vitamin C |  |  |  |  |  |  |  |  |  |  |  |  |  |  |
| T1 | 40,854 | 931/2091 | 1.00 | 299/2091 | 1.00 | 265/2091 | 1.00 | 61,442 | 1419/3179 | 1.00 | 268/3179 | 1.00 | 530/3179 | 1.00 |
| T2 | 18,956 | 352/981 | 0.85 (0.65-1.11) | 82/981 | 0.53 (0.33-0.86) | 102/981 | 0.76 (0.44-1.30) | 82,716 | 1476/4192 | 0.90 (0.76-1.07) | 288/4192 | 0.83 (0.57-1.23) | 552/4192 | 1.01 (0.73-1.41) |
| T3 | 13,282 | 240/737 | 0.88 (0.64-1.20) | 60/737 | 0.68 (0.41-1.12) | 78/737 | 0.96 (0.63-1.47) | 77,805 | 1721/4532 | 0.95 (0.78-1.15) | 310/4532 | 0.80 (0.56-1.15) | 668/4532 | 0.92 (0.64-1.32) |
| P for trend |  |  | 0.340 |  | <0.05 |  | 0.71 |  |  | 0.664 |  | 0.24 |  | 0.59 |
| Potassium |  |  |  |  |  |  |  |  |  |  |  |  |  |  |
| T1 | 22,872 | 472/1218 | 1.00 | 126/1218 | 1.00 | 152/1218 | 1.00 | 87,559 | 1491/4637 | 1.00 | 295/4637 | 1.00 | 551/4637 | 1.00 |
| T2 | 32,917 | 616/1724 | 1.04 (0.79-1.38) | 166/1724 | 1.04 (0.67-1.61) | 175/1724 | 1.16 (0.77-1.75) | 98,215 | 1689/5223 | 0.97 (0.83-1.12) | 324/5223 | 0.85 (0.60-1.20) | 624/5223 | 1.03 (0.80-1.32) |
| T3 | 29,608 | 692/1641 | 1.16 (0.87-1.55) | 219/1641 | 1.33 (0.84-2.11) | 185/1641 | 1.01 (0.63-1.61) | 79,402 | 2222/4715 | 1.12 (0.98-1.27) | 378/4715 | 0.90 (0.65-1.23) | 848/4715 | 1.22 (0.99-1.50) |
| P for trend |  |  | 0.254 |  | 0.18 |  | 0.91 |  |  | 0.050 |  | 0.54 |  | <0.05 |
| Total carotenoids |  |  |  |  |  |  |  |  |  |  |  |  |  |  |
| T1 | 41,179 | 1011/2247 | 1.00 | 310/2247 | 1.00 | 277/2247 | 1.00 | 73,114 | 1855/4136 | 1.00 | 339/4136 | 1.00 | 656/4136 | 1.00 |
| T2 | 28,684 | 491/1490 | 0.57 (0.45-0.72) | 132/1490 | 0.56 (0.38-0.81) | 135/1490 | 0.62 (0.38-1.01) | 92,098 | 1710/4897 | 0.85 (0.73-0.98) | 324/4897 | 0.84 (0.65-1.08) | 668/4897 | 0.97 (0.76-1.24) |
| T3 | 15,534 | 278/846 | 0.66 (0.50-0.86) | 69/846 | 0.59 (0.37-0.95) | 100/846 | 0.86 (0.53-1.40) | 99,964 | 1837/5542 | 0.74 (0.64-0.85) | 334/5542 | 0.54 (0.40-0.74) | 699/5542 | 0.93 (0.72-1.19) |
| P for trend |  |  | <0.0001 |  | <0.01 |  | 0.27 |  |  | <0.0001 |  | <0.0001 |  | 0.54 |
| α-carotene |  |  |  |  |  |  |  |  |  |  |  |  |  |  |
| T1 | 45,902 | 1055/2555 | 1.00 | 309/2555 | 1.00 | 290/2555 | 1.00 | 69,997 | 1536/4016 | 1.00 | 278/4016 | 1.00 | 571/4016 | 1.00 |
| T2 | 24,596 | 458/1286 | 0.83 (0.67-1.03) | 132/1286 | 1.03 (0.73-1.46) | 136/1286 | 0.84 (0.52-1.37) | 84,833 | 1748/4747 | 0.91 (0.81-1.03) | 323/4747 | 0.93 (0.71-1.22) | 655/4747 | 0.85 (0.64-1.13) |
| T3 | 14,899 | 267/742 | 0.65 (0.49-0.85) | 70/742 | 0.70 (0.43-1.14) | 86/742 | 0.71 (0.43-1.17) | 110,346 | 2118/5812 | 0.79 (0.68-0.91) | 396/5812 | 0.75 (0.56-1.00) | 797/5812 | 0.87 (0.64-1.18) |
| P for trend |  |  | <0.01 |  | 0.17 |  | 0.20 |  |  | <0.01 |  | <0.05 |  | 0.40 |
| β-carotene |  |  |  |  |  |  |  |  |  |  |  |  |  |  |
| T1 | 44,753 | 932/2447 | 1.00 | 285/2447 | 1.00 | 266/2447 | 1.00 | 73,327 | 1361/3981 | 1.00 | 268/3981 | 1.00 | 477/3981 | 1.00 |
| T2 | 26,710 | 516/1387 | 0.66 (0.54-0.82) | 136/1387 | 0.66 (0.40-1.07) | 146/1387 | 0.64 (0.39-1.06) | 90,384 | 1747/4922 | 0.82 (0.69-0.97) | 325/4922 | 1.00 (0.74-1.36) | 669/4922 | 0.89 (0.64-1.24) |
| T3 | 13,933 | 332/749 | 0.69 (0.52-0.91) | 90/749 | 0.72 (0.43-1.20) | 100/749 | 0.72 (0.45-1.13) | 101,465 | 2294/5672 | 0.73 (0.63-0.84) | 404/5672 | 0.69 (0.50-0.96) | 877/5672 | 0.86 (0.65-1.15) |
| P for trend |  |  | <0.01 |  | 0.13 |  | 0.096 |  |  | <0.0001 |  | <0.05 |  | 0.32 |
| β-cryptoxanthin |  |  |  |  |  |  |  |  |  |  |  |  |  |  |
| T1 | 38,759 | 938/2217 | 1.00 | 282/2217 | 1.00 | 271/2217 | 1.00 | 60,621 | 1562/3665 | 1.00 | 292/3665 | 1.00 | 574/3665 | 1.00 |
| T2 | 29,832 | 560/1519 | 0.69 (0.53-0.91) | 169/1519 | 0.74 (0.52-1.05) | 150/1519 | 0.66 (0.44-0.99) | 96,213 | 1929/5172 | 0.92 (0.80-1.06) | 369/5172 | 0.89 (0.65-1.21) | 712/5172 | 0.94 (0.75-1.18) |
| T3 | 16,806 | 282/847 | 0.63 (0.48-0.85) | 60/847 | 0.45 (0.24-0.82) | 91/847 | 0.83 (0.51-1.37) | 108,342 | 1911/5738 | 0.77 (0.67-0.89) | 336/5738 | 0.68 (0.48-0.96) | 737/5738 | 0.87 (0.70-1.09) |
| P for trend |  |  | <0.001 |  | <0.01 |  | 0.21 |  |  | <0.001 |  | <0.05 |  | 0.23 |
| Lycopene |  |  |  |  |  |  |  |  |  |  |  |  |  |  |
| T1 | 30,871 | 917/1689 | 1.00 | 255/1689 | 1.00 | 268/1689 | 1.00 | 82,791 | 2572/4689 | 1.00 | 435/4689 | 1.00 | 989/4689 | 1.00 |
| T2 | 29,493 | 531/1460 | 0.77 (0.60-0.99) | 165/1460 | 0.71 (0.48-1.05) | 137/1460 | 0.82 (0.54-1.25) | 97,157 | 1708/4938 | 0.86 (0.76-0.96) | 318/4938 | 0.69 (0.50-0.97) | 629/4938 | 0.88 (0.69-1.12) |
| T3 | 25,032 | 332/1434 | 0.67 (0.51-0.87) | 91/1434 | 0.72 (0.48-1.08) | 107/1434 | 0.75 (0.43-1.30) | 85,228 | 1122/4948 | 0.84 (0.72-0.97) | 244/4948 | 0.73 (0.53-1.00) | 405/4948 | 0.88 (0.68-1.14) |
| P for trend |  |  | <0.01 |  | 0.090 |  | 0.28 |  |  | <0.05 |  | <0.05 |  | 0.28 |
| Lutein & zeaxanthin |  |  |  |  |  |  |  |  |  |  |  |  |  |  |
| T1 | 34,042 | 751/2001 | 1.00 | 219/2001 | 1.00 | 209/2001 | 1.00 | 72,664 | 1526/4391 | 1.00 | 268/4391 | 1.00 | 537/4391 | 1.00 |
| T2 | 27,698 | 530/1428 | 0.71 (0.57-0.89) | 156/1428 | 0.61 (0.44-0.84) | 149/1428 | 0.77 (0.50-1.18) | 90430 | 1728/4949 | 0.93 (0.80-1.07) | 346/4949 | 1.09 (0.76-1.56) | 618/4949 | 0.93 (0.70-1.24) |
| T3 | 23,657 | 499/1154 | 0.65 (0.53-0.80) | 136/1154 | 0.70 (0.46-1.06) | 154/1154 | 0.92 (0.60-1.42) | 102,082 | 2148/5235 | 0.82 (0.71-0.94) | 383/5235 | 0.85 (0.62-1.17) | 868/5235 | 1.00 (0.78-1.29) |
| P for trend |  |  | <0.0001 |  | 0.060 |  | 0.65 |  |  | <0.01 |  | 0.19 |  | 0.87 |

^*^ HRs and 95% CIs were adjusted for age, ethnicity, energy intake, BMI, PIR, diabetes, hypertension, alcohol consumption, saturated fatty acid intake, aspirin use, supplement use, CKD and history of CVD; excluding history of CVD for CVD death.
